# Supplementary material for: The selection of a hydrophobic 7-phenylbutyl-7-deazaadenine-modified DNA aptamer with high binding affinity for the Heat Shock Protein 70
Source: Commun Chem. 2023 Apr 6;6:65. doi: 10.1038/s42004-023-00862-0 (PMC10079658; doi:10.1038/s42004-023-00862-0)
Supplement: Supplementary file 2 — Supplementary Information [file 42004_2023_862_MOESM2_ESM.pdf]

Supporting Information

The selection of a hydrophobic 7-phenylbutyl-7-deazaadenine-modified DNA aptamer with high binding affinity for the Heat Shock Protein 70

Contents

1. Experimental section – Organic Chemistry 2

1.1 General Remarks 2

1.2 Chemical synthesis 3

1.3 Oligonucleotide synthesis 10

1.4 NMR Spectra 12

1.5 MALDI-TOF Mass Spectra 20

2. Experimental section – Biochemistry 23

2.1 General Remarks 23

2.2 Cloning and protein purification of His-tagged Hsp70 24

2.3 The use of modified dA*TP for incorporation into DNA by PEX 25

2.4 Example of gel electrophoresis of amplified 65 bp product (from round 1) 26

2.5 Example of gel electrophoresis of ssDNA generation (from round 1) 27

2.6 Identification of aptamer sequences 28

2.7 Enzymatic synthesis and initial screening of candidates HSe-1 to HSe-9 29

2.8 In-house chemical synthesis of aptamers HSc-2 and HSc-9 31

2.9 BLI steady state raw data files for modified aptamer HSc-2 32

2.10 BLI steady state raw data files for natural aptamer HSNat-2 33

2.11 BLI steady state raw data files for modified aptamer HSc-9 34

2.12 BLI steady state raw data files for natural aptamer HSNat-9 35

2.13 BLI steady state raw data files for modified aptamer HSc-9.1 36

2.14 3'-Biotinylated HSc-9.1 as a capture aptamer for Hsp70 detection by anti-Hsp70 antibody in a sandwich-based ELISA 37

2.15 Specificity of HSc-9.1 aptamer against His-tagged proteins 40

2.16 Specificity of HSc-9.1 aptamer against Streptavidin binding peptide (SBP)-tagged Hsp70 41

2.17 Specificity of scrambled HSc-9 aptamer against His-tagged Hsp-70 41

Supplementary References 43

Supplementary Methods

1. Experimental section – Organic Chemistry

1.1 General Remarks

All chemicals used were purchased from common commercial suppliers, such as Fluorochem and Sigma Aldrich. Reactions were monitored by silica gel thin-layer chromatography (TLC) in Merck silica gel 60 F_{254} plates with UV light detection (254 and 365 nm) combined with visualization by the solution of 4-anisaldehyde in ethanol with sulphuric acid (10%). The masses of individual spots on TLC plate were measured by Advion Expression Compact Mass Spectrometer connected with Plate Express® TLC Plate Reader (TLC-MS) using electrospray ionization (ESI). Solvents were removed *in vacuo* with the bath temperature between 40–60 °C. Purification of nucleosides was carried out on CombiFlash Rf+ (Teledyne Isco) with columns filled with Silicagel 40–63 μm from VWR International. Purity of all final compounds was determined by NMR and MS spectra. ^{1}H and ^{13}C NMR spectra were measured on Bruker Avance III^{TM} HD 500 MHz (^{1}H at 500.0 MHz, ^{13}C at 125.7 MHz and ^{31}P at 202.4 MHz) and JEOL ECZR 500 MHz (^{1}H at 500.2 MHz, ^{13}C at 125.8 MHz and ^{31}P at 202.5 MHz) in DMSO-*d6*, D_2O or CD_3CN referenced to the residual solvent signal. Chemical shifts are given in ppm (δ-scale), coupling constants (J) in Hz. Complete assignment of all NMR signals was performed using a combination of H,H-COSY, H,C-HSQC and H,C-HMBC experiments. MestreNova (version 14) from Mestrelab Research was used for data evaluation. Low- and high-resolution mass spectra were measured on LTQ Orbitrap XL spectrometer (ESI ionization, Thermo Fisher Scientific). The MALDI-TOF spectra were measured on a UltrafleXtreme MALDI-TOF/TOF (Bruker) mass spectrometer. The matrix consisted of 3-hydroxypicolinic acid (HPA)/picolinic acid (PA)/ ammonium tartrate in ratio 9:1:1. The matrix (1 μL) was applied to the target (ground steel) and dried down at room temperature. All mass spectra were acquired by the MS service at IOCB.

Reagents and solvents for the solid-phase synthesis of oligonucleotides were purchased from Sigma-Aldrich, Link Technologies, and Thermo Fisher-Scientifics. Modified oligonucleotides were synthesized through standard phosphoramidite chemistry with an automated DNA synthesizer (Mermade 8, Bioautomation Corporation). Purification of the prepared oligonucleotides was performed using semi-preparative HPLC (Waters modular HPLC system)

on a column packed with 10 μm C18 reversed phase (Phenomenex, Kinetex 5 μm EVO C18 100 Å). The products were analyzed by MALDI-TOF MS and ESI-MS. Concentrations of DNA solutions were calculated using extinction coefficients acquired from the on-line tool at https://www.atdbio.com/tools/oligo-calculator and A260 values measured on a Cary100 Bio UV-Vis spectrophotometer (Varian). The chemical synthesis of all products and oligos are provided in supplementary information.

1.2 Chemical synthesis

4-Chloro-5-iodo-7*H*-pyrrolo[2,3-*d*]pyrimidine

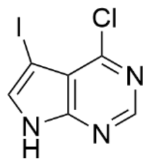

4-Chloro-7*H*-pyrrolo[2,3-*d*]pyrimidine (14 g, 91.2 mmol) and *N*-iodosuccinimide (21.5 g, 95.8 mmol) were dissolved in 120 mL of dry DMF and left stirring at room temperature in the darkness overnight. The reaction mixture was then poured into ice-cold water (800 mL) and the precipitate was filtered off to afford 24.6 g of desired compound (97%). NMR and MS spectra were in accord with literature.[1]

3′,5′-Bis(4-chlorobenzoyl) 7-iodo-2′-deoxy-7-deazaadenosine

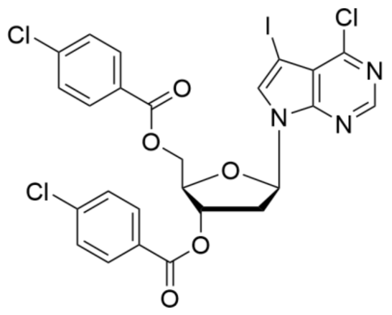

To a solution of 4-chloro-5-iodo-7*H*-pyrrolo[2.3-*d*]pyrimidine (11.8 g, 42.4 mmol) in dry MeCN (600 mL), powdered KOH (5.9 g, 105.6 mmol) was added at room temperature, the mixture was stirred for 3 min and TDA-1 (1.0 mL, 3.0 mmol) was subsequently injected. After stirring for 20 min, 3,5-*O*-bis(4-chlorobenzoyl)-2-deoxy-α-D-ribofuranosyl chloride (23.6 g, 54.9 mmol) was added and the stirring was continued for 40 min. After total conversion monitored by TLC analysis (DCM/MeOH 40:1), saturated solution of NH_4Cl (200 mL) was added to precipitate the product. Subsequently the precipitated solid was filtered off, washed with water (200 mL) and acetone (200 mL) and dried to give desired product (49.9 g, 88%). NMR and MS spectra were in agreement with literature.[1]

7-Iodo-2′-deoxy-7-deazaadenosine (1, dA^{I})

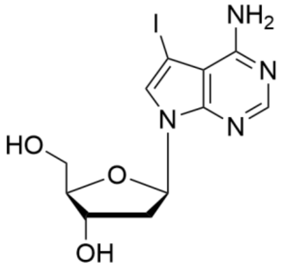

A solution of compound **4** (14 g, 20.8 mmol) in NH_3 (aq.)/dioxane (200 mL, 1:1 [*v:v*]) was stirred at 110 °C for 2 days and the solvent was subsequently evaporated under reduced pressure. Crystallization from ethanol yielded compound **1** (**dA^{I}**, 4.7 g, 67%) as a brownish solid. NMR and MS spectra were in accord with literature[1].

7-(4-Phenylbut-1-yn-1-yl)-2′-deoxy-7-deazaadenosine (2, dA^{EEPh})

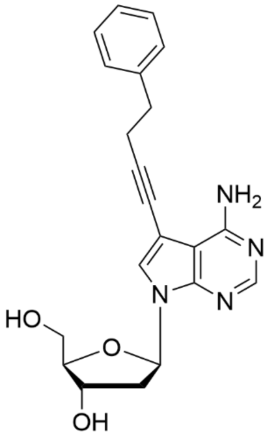

Mixture of MeCN/H_2O (1:1, 300 mL) was added through a septum to an argon-purged flask containing compound **1** (**dA^{I}**, 2.5 g, 6.7 mmol), $Pd(OAc)_2$ (149 mg, 0.7 mmol), TPPTS (378 mg, 0.7 mmol) and CuI (127 mg, 0.7 mmol). Then the but-3-yn-1-ylbenzene (9.3 mL, 66.5 mmol) was added followed by addition of TEA (5.6 mL, 39.9 mmol). The mixture was stirred at room temperature overnight and the solvent was removed under reduced pressure. The product was purified by HPFC chromatography (DCM/MeOH 0–10%) followed by evaporation under vacuum, sonication with H_2O/EtOH (to remove TEA) and co-evaporation with DCM to get pure product **2** (**dA^{EEPh}**) as a white solid (2.4 g, 93%).

^{1}H NMR (500.0 MHz, DMSO-d_6): 2.16 (ddd, 1H, J_{gem} = 13.1, $J_{2'b,1'}$ = 6.0, $J_{2'b,3'}$ = 2.7, H-2′b); 2.45 (ddd, 1H, J_{gem} = 13.1, $J_{2'a,1'}$ = 8.1, $J_{2'a,3'}$ = 5.7, H-2′a); 2.76–2.80 (m, 2H, C**H$_2$**CH$_2$Ph); 2.85–2.89 (m, 2H, CH$_2$C**H$_2$**Ph); 3.50 (ddd, 1H, J_{gem} = 11.7, $J_{5'b,OH}$ = 5.5, $J_{5'b,4'}$ = 4.2, H-5′b); 3.57 (ddd, 1H, J_{gem} = 11.7, $J_{5'a,OH}$ = 5.5, $J_{5'a,4'}$ = 4.6, H-5′a); 3.82 (ddd, 1H, $J_{4',5'}$ = 4.6, 4.2, $J_{4',3'}$ = 2.5, H-4′); 4.33 (m, 1H, H-3′); 5.07 (t, 1H, $J_{OH,5'}$ = 5.5, OH-5′); 5.26 (d, 1H, $J_{OH,3'}$ = 4.1, OH-3′); 6.46 (dd, 1H, $J_{1',2'}$ = 8.1, 6.0, H-1′); 7.21 (m, 1H, H-*p*-Ph); 7.28–7.34 (m, 2H, H-*o,m*-Ph); 7.63 (s, 1H, H-6); 8.09 (s, 1H, H-2).

^{13}C NMR (125.7 MHz, DMSO-d6): 21.17 (**C**H$_2$CH$_2$Ph); 34.36 (CH$_2$**C**H$_2$Ph); 40.00 (CH$_2$-2′); 62.08 (CH$_2$-5′); 71.17 (CH-3′); 74.32 (**C**≡C-CH$_2$); 83.33 (CH-1′); 87.67 (CH-4′); 92.11 (C≡**C**-CH$_2$); 95.54 (C-5); 102.37 (C-4a); 125.81 (CH-6); 126.47 (CH-*p*-Ph); 128.56, 128.57 (CH-*o,m*-Ph); 140.70 (C-*i*-Ph); 149.20 (C-7a); 152.75 (CH-2); 157.67 (C-4).

HR-ESI-MS calculated m/z: 379.17647 $[M+H]^+$, 401.15841 $[M+Na]^+$, found m/z: 379.17616 $[M+H]^+$, 401.15817 $[M+Na]^+$.

7-(4-Phenylbutyl)-2′-deoxy-7-deazaadenosine (3, dA$^{\mathbf{BuPh}}$)

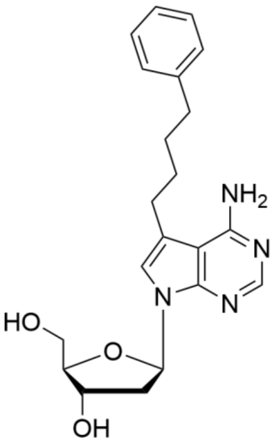

To an argon-purged flask containing corresponding precursor **2** (4.1 g, 10.9 mmol) and 10% Pd/C (1.2 g, 1.1 mmol), MeOH (160 mL) was added through a septum followed by fulfilling the flask with H_2 atmosphere (baloons). The reaction mixture was stirred at room temperature until complete consumption of the starting material. The solvent was evaporated under vacuum and the residue was purified by HPFC chromatography (DCM/MeOH 0–15%) followed by solvent evaporation to obtain product **3** (**dA$^{\mathbf{BuPh}}$**) as a white solid (3.2 g, 77%).

^{1}H NMR (500.2 MHz, DMSO-d_6): 1.52–1.64 (m, 2H, $CH_2C\mathbf{H_2}CH_2CH_2Ph$); 1.59–1.71 (m, 1H, $CH_2CH_2C\mathbf{H_2}CH_2Ph$); 2.16 (ddd, 1H, J_{gem} = 13.1, $J_{2'b,1'}$ = 6.0, $J_{2'b,3'}$ = 2.6, H-2′b); 2.44 (ddd, 1H, J_{gem} = 13.1, $J_{2'a,1'}$ = 8.1, $J_{2'a,3'}$ = 5.8, H-2′a); 2.59–2.63 (m, 2H, $CH_2CH_2CH_2C\mathbf{H_2}Ph$); 2.77–2.82 (m, 2H, $C\mathbf{H_2}CH_2CH_2CH_2Ph$); 3.49 (dd, 1H, J_{gem} = 11.6, $J_{5'b,4'}$ = 4.4, H-5′b); 3.56 (dd, 1H, J_{gem} = 11.6, $J_{5'a,4'}$ = 4.7, H-5′a); 3.81 (ddd, 1H, $J_{4',5'}$ = 4.7, 4.4, $J_{4',3'}$ = 2.6, H-4′); 4.33 (dt, 1H, $J_{3',2'}$ = 5.8, 2.6, $J_{3',4'}$ = 2.6, H-3′); 5.28 (bs, 1H, OH-3′); 6.50 (dd, 1H, $J_{1',2'}$ = 8.1, 6.0, H-1′); 7.15 (m, 1H, H-*p*-Ph); 7.17–7.20 (m, 2H, H-*o*-Ph); 7.24–7.28 (m, 2H, H-*m*-Ph); 7.31 (s, 1H, H-6); 7.47 (bs, 2H, NH_2); 8.19 (s, 1H, H-2).

^{13}C NMR (125.8 MHz, DMSO-d_6): 25.40 ($\mathbf{C}H_2CH_2CH_2CH_2Ph$); 29.83 ($CH_2\mathbf{C}H_2CH_2CH_2Ph$); 30.58 ($CH_2CH_2\mathbf{C}H_2CH_2Ph$); 35.10 ($CH_2CH_2CH_2\mathbf{C}H_2Ph$); 39.87 ($CH_2$-2′); 62.15 ($CH_2$-5′); 71.20 (CH-3′); 83.06 (CH-1′); 87.61 (CH-4′); 101.50 (C-4a); 117.14 (C-5); 120.43 (CH-6); 125.84 (CH-*p*-Ph); 128.46, 128.49 (CH-*o,m*-Ph); 142.49 (C-*i*-Ph); 146.90 (CH-2); 148.96 (C-7a); 154.27 (C-4).

HR-ESI-MS calculated m/z: 383.20777 $[M+H]^+$, 405.18971 $[M+Na]^+$, found m/z: 383.20763 $[M+H]^+$, 405.18960 $[M+Na]^+$.

7-(4-Phenylbutyl)-2′-deoxy-7-deazaadenosine 5′-*O*-triphosphate (4, dA^{BuPh}TP)

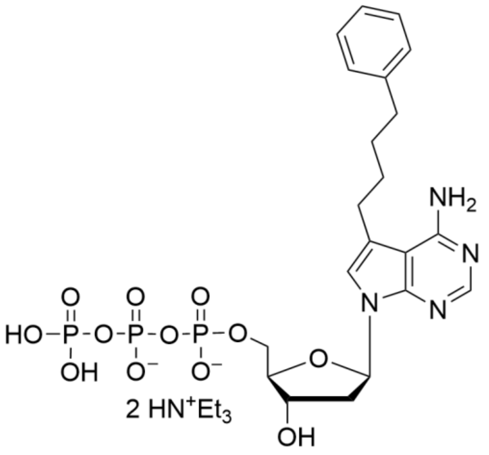

Distilled $PO(OMe)_3$ (1 mL) was added through a septum to an argon-purged flask containing compound **3** (**dA^{BuPh}**, 50 mg, 0.13 mmol) previously dried by co-evaporation with anhydrous pyridine (2×5 mL) and DCM (3×5 mL) followed by drying under vacuum at 65 °C for 1 hour. After the dropwise addition of distilled $POCl_3$ (1.2 equiv., 15 *μ*l, 0.16 mmol) at 0 °C, the reaction mixture was stirred for 2 hours at 0 °C (TLC analysis in DCM/MeOH 10:1). Subsequently, ice-cooled mixture of $(NHBu_3)_2H_2P_2O_7$ (5 equiv., 359 mg, 0.65 mmol) and Bu_3N (4 equiv., 124 *μ*l, 0.52 mmol) in dry MeCN (2 mL) was added dropwise. The reaction mixture was then stirred for another 1 hour at 0 °C under inert atmosphere (TLC analysis in isopropylalcohol/ammonia (aq.)/water 11:2:7) and subsequently slowly stopped by aqueous solution of 2 M TEAB (triethylammonium bicarbonate, 5 mL). Solvents were then evaporated under reduced pressure and the product was purified by HPLC on a C18 column with use of linear gradient from 0.1 M TEAB in H_2O to 0.1 M TEAB in H_2O/MeOH (1:1). After evaporation of the solvent and several co-evaporations with H_2O, the lyofilization from water afforded the triphosphate **4** (15 mg, 19%).

^{1}H NMR (500.0 MHz, CD_3OD): 1.30 (t, 18H, J_{vic} = 7.3, C**H₃**CH₂N); 1.55–1.63 (m, 2H, CH₂C**H₂**CH₂CH₂Ph); 1.63–1.71 (m, 1H, CH₂CH₂C**H₂**CH₂Ph); 2.25 (ddd, 1H, J_{gem} = 13.6, $J_{2'b,1'}$ = 6.1, $J_{2'b,3'}$ = 3.2, H-2′b); 2.44 (ddd, 1H, J_{gem} = 13.6, $J_{2'a,1'}$ = 7.9, $J_{2'a,3'}$ = 6.1, H-2′a); 2.59–2.64 (m, 2H, CH₂CH₂CH₂C**H₂**Ph); 2.64–2.68 (bm, 2H, C**H₂**CH₂CH₂CH₂Ph); 3.19 (q, 12H, J_{vic} = 7.3, CH₃C**H₂**N); 4.13 (qd, 1H, $J_{4',3'}$ = $J_{4',5'}$ = 3.8, $J_{H,P}$ = 1.0, H-4′); 4.20–4.28 (m, 2H, H-5′); 4.60 (dt, 1H, $J_{3',2'}$ = 6.1, 3.2, $J_{3',4'}$ = 3.2, H-3′); 6.57 (dd, 1H, $J_{1',2'}$ = 7.9, 6.1, H-1′); 7.11 (m, 1H, H-*p*-Ph); 7.14–7.17 (m, 2H, H-*o*-Ph); 7.19–7.23 (m, 2H, H-*m*-Ph); 7.29 (s, 1H, H-6); 8.15 (s, 1H, H-2).

^{13}C NMR (125.7 MHz, CD_3OD): 9.13 (**C**H₃CH₂N); 26.91 (**C**H₂CH₂CH₂CH₂Ph); 31.38 (CH₂**C**H₂CH₂CH₂Ph); 32.21 (CH₂CH₂**C**H₂CH₂Ph); 36.70 (CH₂CH₂CH₂**C**H₂Ph); 41.34 (CH₂-2′); 47.51 (CH₃**C**H₂N); 67.00 (d, $J_{C,P}$ = 5.4, CH₂-5′); 72.67 (CH-3′); 84.32 (CH-1′); 87.22 (d, $J_{C,P}$ = 8.6, CH-4′); 102.20 (C-4a); 119.51 (C-5); 121.79 (CH-6); 126.62 (CH-*p*-Ph); 129.27 (CH-*m*-Ph); 129.47 (CH-*o*-Ph); 143.93 (C-*i*-Ph); 146.51 (CH-2); 149.71 (C-7a); 154.35 (C-4).

$^{31}P\{^{1}H\}$ NMR (202.4 MHz, CD_3OD): -21.44 (bdd, *J* = 20.3, 19.1, $P_□$); -9.28 (d, *J* = 20.3, $P_□$); -8.41 (d, *J* = 19.1, $P_□$).

HR-ESI-MS calculated for $C_{21}H_{28}O_{12}N_4P_3$ m/z: 621.09220 $[M+3H]^-$, found m/z: 621.09186 $[M+3H]^-$.

5'-*O*-[Bis(4-methoxyphenyl)phenylmethyl]-7-(4-phenylbutyl)-2′-deoxy-7-deazaadenosine (5)

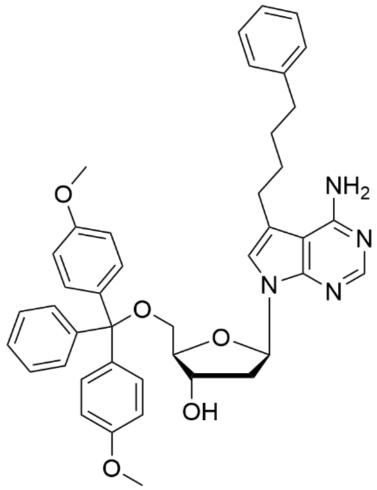

Precursor **3** (4 g, 10.5 mmol) was dried by several co-evaporations with anhydrous pyridine (3×5 mL) and finally dissolved in 40 mL of anhydrous pyridine along with DMAP (117 mg, 1.1 mmol). Solution of 4,4'-dimethoxytrityl chloride (DMTrCl, 4.3 g, 12.6 mmol) in 20 mL of anhydrous pyridine was added in 4 portions over 1 hour and the reaction mixture was stirred at room temperature overnight. The solvent was removed under reduced pressure at a rotary evaporator and the crude reaction mixture was re-dissolved in DCM, washed with 10% aqueous solution of $NaHCO_3$ (200 mL), brine (sat., 200 mL), and finally dried over anhydrous Na_2SO_4 for 20 min. Purification by HPFC chromatography (DCM/MeOH 0–1% with 0.5% TEA) afforded the desired compound **5** as a yellowish solid (3.8 g, 53%).

^{1}H NMR (500.2 MHz, DMSO-d_6): 1.37–1.46 (m, 2H, $CH_2C\mathbf{H_2}CH_2CH_2Ph$); 1.49–1.58 (m, 2H, $CH_2CH_2C\mathbf{H_2}CH_2Ph$); 2.21 (ddd, 1H, J_{gem} = 13.2 hz, $J_{2'b,1'}$ = 6.2 hz, $J_{2'b,3'}$ = 3.4 hz, H-2´a); 2.43–2.53 (m, 3H, H-2´b, $CH_2CH_2CH_2C\mathbf{H_2}Ph$); 2.58–2.72 (m, 2H, $C\mathbf{H_2}CH_2CH_2CH_2Ph$); 3.11–3.19 (m, 2H, H-5´); 3.71 (s, 2 × 3H, CH_3O-DMTr); 3.91 (td, 1H, $J_{4',5'b} = J_{4',5'a}$ = 4.2 hz, $J_{4',3'}$ = 3.4 hz, H-4´); 4.40 (m, 1H, H-3´); 5.32 (d, 1H, $J_{OH,3'}$ = 4.4 hz, OH-3´); 6.54 (dd, 1H, $J_{1',2'b}$ = 7.4 hz, $J_{1',2'a}$ = 6.3 hz, H-1´); 6.53 (bs, 2H, NH_2); 6.80–6.87 (m, 4H, H-*m*-C_6H_4OMe-DMTr); 6.94 (s, 1H, H-6); 7.08–7.16 (m, 3H, H-*o,p*-Ph); 7.17–7.29 (m, 9H, H-*m*-PH, H-*m,p*-C_6H_5-DMTr, H-*o*-C_6H_4OMe-DMTr); 7.36–7.40 (m, 2H, H-*o*-C_6H_5-DMTr); 8.02 (s, 1H, H-2).

^{13}C NMR (125.8 MHz, DMSO-d_6): 25.76 ($\mathbf{C}H_2CH_2CH_2CH_2Ph$); 29.98 ($CH_2\mathbf{C}H_2CH_2CH_2Ph$); 30.68 ($CH_2CH_2\mathbf{C}H_2CH_2Ph$); 35.07 ($CH_2CH_2CH_2\mathbf{C}H_2Ph$); 39.7 (*overlapped with DMSO*, CH_2-2´); 55.20 (CH_3O-DMTr); 64.31 (CH_2-5´); 71.19 (CH-3´); 82.29 (CH-1´); 85.33 (CH-4´); 85.79

(C-DMTr); 102.11 (C-4a); 113.32 (CH-*m*-C_6H_4OMe-DMTr); 115.86 (C-5); 118.05 (CH-6); 125.74 (CH-*p*-Ph); 126.87 (CH-*p*-C_6H_5-DMTr); 127.98 (CH-*o*,*m*-C_6H_5-DMTr); 128.36 and 128.38 (CH-*o*,*m*-Ph); 129.92 and 129.94 (CH-*o*-C_6H_4OMe-DMTr); 135.73 and 135.80 (C-*i*-C_6H_4OMe-DMTr); 142.47 (C-*i*-Ph); 144.98 (C-*i*-C_6H_5-DMTr); 150.72 (C-7a); 151.56 (CH-2); 157.70 (C-4); 158.26 (C-*p*-C_6H_4OMe-DMTr).

HR-ESI-MS calculated m/z: 685.33845 $[M+H]^+$, 707.32039 $[M+Na]^+$, found m/z: 685.33814 $[M+H]^+$, 707.32004 $[M+Na]^+$.

N^6-Dimethylformimidamide-5'-*O*-[bis(4-methoxyphenyl)phenylmethyl]-7-(4-phenylbutyl)-2′-deoxy-7-deazaadenosine (6)

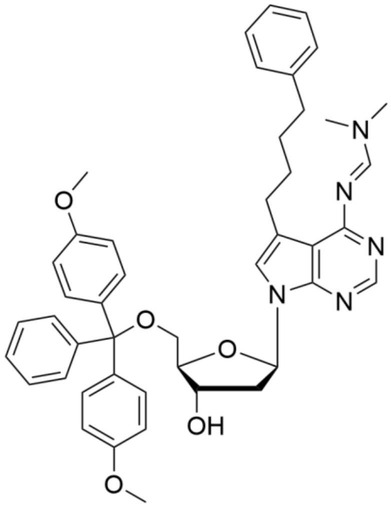

To the compound **5** (2.6 g, 3.84 mmol) dissolved in anhydrous DMF (50 mL) under argon atmosphere, dimethylformamide dimethylacetal (DMF-DMA, 7.2 mL, 53.75 mmol) was added and the reaction was stirred 4 hours at 40 °C. After disappearance of the starting material (monitored by TLC, DCM/MeOH 20:1) DCM was added, and the organic layer was washed twice with saturated $NaHCO_3$ (200 mL), followed by brine (200 mL) and dried over Na_2SO_4 for 20 min. The crude product was then isolated by HPFC chromatography (DCM/MeOH 0–1.2% with 0.5% TEA). The solvents were removed under vaccum and the product **6** was obtained as a white foam (2.3 g, 81%).

^{1}H NMR (500.2 MHz, DMSO-d_6): 1.48–1.61 (m, 4H, $CH_2C\mathbf{H_2}C\mathbf{H_2}CH_2$Ph); 2.23 (ddd, 1H, J_{gem} = 13.2 hz, $J_{2´b,1´}$ = 6.4 hz, $J_{2´b,3´}$ = 3.6 hz, H-2´a); 2.54 (ddd, 1H, J_{gem} = 13.1 hz, $J_{2´b,1´}$ = 7.3 hz, $J_{2´b,3´}$ = 6.2 hz, H-2´b); 2.44–2.54 (m, 2H, $CH_2CH_2CH_2C\mathbf{H_2}$Ph); 2.67–2.74 (m, 2H, $C\mathbf{H_2}CH_2CH_2CH_2$Ph); 3.04 (s, 3H, $(CH_3)_2N$); 3.14 (dd, 1H, J_{gem} = 10.2 hz, $J_{5´a,4'}$ = 3.7 hz, H-5´a); 3.15 (s, 3H, $(CH_3)_2N$); 3.17 (dd, 1H, J_{gem} = 10.3 hz, $J_{5´b,4´}$ = 5.0 hz, H-5´b); 3.709 and 3.711 (2 × s, 2 × 3H, CH_3O-DMTr); 3.93 (dt, 1H, $J_{4´,5´b}$ = 4.9 hz, $J_{4´,5´a}$ = $J_{4´,3´}$ = 3.6 hz, H-4´); 4.43 (m, 1H, H-3´); 5.34 (d, 1H, $J_{OH,3´}$ = 4.4 hz, OH-3´); 6.58 (dd, 1H, $J_{1´,2´b}$ = 7.3 hz, $J_{1´,2´a}$ = 6.3 hz, H-1´); 6.80–6.86 (m, 4H, H-*m*-C_6H_4OMe-DMTr); 7.08 (s, 1H, H-6); 7.06–7.11 (m, 2H, H-*o*-Ph); 7.13 (m, 1H, H-*p*-Ph); 7.17–7.29 (m, 9H, H-*m*-PH, H-*m,p*-C_6H_5-DMTr, H-*o*-C_6H_4OMe-DMTr); 7.36–7.40 (m, 2H, H-*o*-C_6H_5-DMTr); 8.26 (s, 1H, H-2); 8.81 (s, 1H, NCH=N).

^{13}C NMR (125.8 MHz, DMSO-d_6): 26.51 (**C**$H_2CH_2CH_2CH_2$Ph); 30.89 and 31.44 (CH_2**C**H_2**C**H_2CH_2Ph); 34.75 ($(CH_3)_2N$); 35.37 ($CH_2CH_2CH_2$**C**H_2Ph); 39.95 (CH_2-2´); 40.52 ($(CH_3)_2N$); 55.18 (CH_3O-DMTr); 64.35 (CH_2-5´); 71.19 (CH-3´); 82.35 (CH-1´); 85.37 (CH-4´); 85.80 (C-DMTr); 110.11 (C-4a); 113.31 (CH-*m*-C_6H_4OMe-DMTr); 117.26 (C-5); 119.91 (CH-6); 125.73 (CH-*p*-Ph); 126.86 (CH-*p*-C_6H_5-DMTr); 127.98 (CH-*o*,*m*-C_6H_5-DMTr); 128.38 (CH-*o*,*m*-Ph); 129.92 and 129.97 (CH-*o*-C_6H_4OMe-DMTr); 135.72 and 135.79 (C-*i*-C_6H_4OMe-DMTr); 142.53 (C-*i*-Ph); 145.00 (C-*i*-C_6H_5-DMTr); 151.09 (CH-2); 152.02 (C-7a); 156.43 (NCH=N); 158.24 and 158.26 (C-*p*-C_6H_4OMe-DMTr); 160.75 (C-4).

HR-ESI-MS calculated m/z: 740.38065 $[M+H]^+$, 762.36259 $[M+Na]^+$, found m/z: 740.38041 $[M+H]^+$, 762.36245 $[M+Na]^+$.

N^6-Dimethylaminomethylene-5'-*O*-[bis(4-methoxyphenyl)phenylmethyl]-7-(4-phenylbutyl)-2′-deoxy-7-deazaadenosine-3′-(2-cyanoethyl *N,N*-diisopropylphosphoramidite) (7)

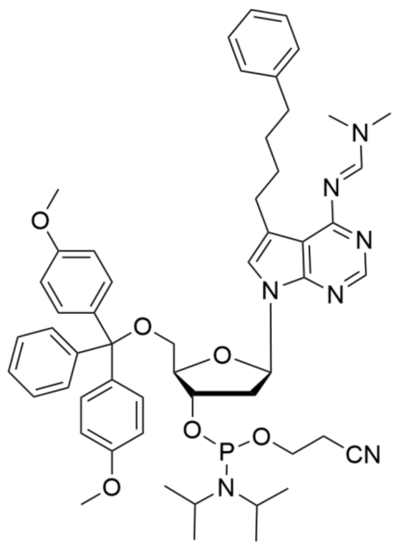

Protected nucleoside **6** (2.3 g, 3.11 mmol) was dried by repeated co-evaporation with anhydrous pyridine (3×5 mL), followed by co-evaporation with anhydrous DCM (3×5 mL), and dried under vacuum for 30 min and subsequently, the starting material was dissolved in anhydrous DCM. After freshly distilled *N*,*N*-diisopropylethylamine (DIPEA, 1.4 mL, 7.77 mmol) was added, the reaction mixture was cooled down to 0 °C and 2-cyanoethyl-*N*,*N*-diisopropylchlorophosphoramidite (883 mg, 3.73 mmol) was injected. The reaction mixture was allowed to warm up to room temperature and was stirred until a complete conversion was observed by TLC analysis (cyclohexane/EtOAc 1:4, approx. 1.5 h). Then, the reaction mixture was diluted with anhydrous DCM, quickly washed under an argon atmosphere with saturated aqueous KI (20 mL) and dried over Na_2SO_4. Purification by HPFC chromatography (cyclohexane/EtOAc 2:1 to 100% EtOAc with 0.5% TEA) under argon atmosphere provided final compound **7** as two diastereomers (5:2, 2.5 g, 86%).

^{1}H NMR (500.0 MHz, CD_3CN): 1.17 and 1.18 (2×d, 2×6H, $J_{CH3,CH}$ = 6.7 hz, (C**H$_3$**)$_2$CHN); 1.52–1.66 (m, 4H, CH_2C**H$_2$**C**H$_2$**CH_2Ph); 2.44 (ddd, 1H, J_{gem} = 13.5 hz, $J_{2´b,1´}$ = 6.4 hz, $J_{2´b,3´}$ =

4.2 hz, H-2´a); 2.50–2.56 (m, 4H, $CH_2CH_2CH_2C\mathbf{H_2}PH$, $OCH_2C\mathbf{H_2}CN$); 2.70–2.80 (m 3H, H-2´b, $C\mathbf{H_2}CH_2CH_2CH_2Ph$); 3.04 and 3.13 (2×s, 2×3H, $(CH_3)_2N$); 3.22 (dd, 1H, J_{gem} = 10.4 hz, $J_{5'a,4'}$ = 3.4 hz, H-5´a); 3.28 (dd, 1H, J_{gem} = 10.4 hz, $J_{5'b,4'}$ = 4.5 hz, H-5´b); 3.57–3.73 (m, 4H, $(CH_3)_2C\mathbf{H}N$, $OC\mathbf{H_2}CH_2CN$), 3.74 (s, 2×3H, CH_3O-DMTr); 4.11 (m, 1H, H-4´); 4.80 (ddt, 1H, $J_{3',P}$ = 10.8 hz, $J_{3',1'}$ = 6.4 hz, $J_{3',2'a}$ = $J_{3',2'b}$ = 4.0 hz, H-3´); 6.59 (t, 1H, $J_{1',2'b}$ = $J_{1',2'a}$ = 6.7 hz, H-1´); 6.77–6.82 (m, 4H, H-*m*-C_6H_4OMe-DMTr); 7.01 (s, 1H, H-6); 7.09–7.15 (m, 3H, H-*o,p*-Ph); 7.18–7.27 (m, 5H, H-*m*-PH, H-*m,p*-C_6H_5-DMTr); 7.27–7.32 (m, 4H, H-*o*-C_6H_4OMe-DMTr); 7.41–7.44 (m, 2H, H-*o*-C_6H_5-DMTr); 8.27 (s, 1H, H-2); 8.77 (s, 1H, NCH=N).

^{13}C NMR (125.7 MHz, CD_3CN): 20.98 (d, $J_{C,P}$ = 7.0 hz, $OCH_2\mathbf{C}H_2CN$); 24.87 and 24.93 ($(\mathbf{C}H_3)_2CHN$); 27.44 ($\mathbf{C}H_2CH_2CH_2CH_2Ph$); 31.92 ($CH_2\mathbf{C}H_2CH_2CH_2Ph$); 32.43 ($CH_2CH_2\mathbf{C}H_2CH_2Ph$); 35.09 ($(CH_3)_2N$); 36.43 ($CH_2CH_2CH_2\mathbf{C}H_2Ph$); 39.72 (d, $J_{C,P}$ = 4.3 hz, CH_2-2´); 41.15 ($(CH_3)_2N$); 43.99 ($(CH_3)_2\mathbf{C}HN$); 55.88 (CH_3O-DMTr); 59.49 (d, $J_{C,P}$ = 18.9 hz, $O\mathbf{C}H_2CH_2CN$); 64.49 (CH_2-5´); 74.26 (d, $J_{C,P}$ = 16.5 hz, CH-3´); 83.66 (CH-1´); 85.51 (d, $J_{C,P}$ = 4.2 hz, CH-4´); 87.11 (C-DMTr); 111.46 (C-4a); 113.98 (CH-*m*-C_6H_4OMe-DMTr); 118.98 (C-5); 119.42 ($OCH_2CH_2\mathbf{C}N$); 120.70 (CH-6); 126.50 (CH-*p*-Ph); 127.82 (CH-*m*-C_6H_5-DMTr); 128.78, 129.13, 129.19 (CH-*o,p*-C_6H_5-DMTr, CH-*m*-Ph); 129.33 (CH-*o*-Ph); 131.05 and 131.07 (CH-*o*-C_6H_4OMe-DMTr); 136.92 (C-*i*-C_6H_4OMe-DMTr); 143.94 (C-*i*-Ph); 146.04 (C-*i*-C_6H_5-DMTr); 152.31 (CH-2); 153.41 (C-7a); 157.21 (NCH=N); 159.62 and 158.63 (C-*p*-C_6H_4OMe-DMTr); 162.12 (C-4).

^{31}P NMR (202.4 MHz, CD_3CN): 149.14 (s, 1P).

HR-ESI-MS calculated m/z: 940.48850 $[M+H]^+$, 962.47044 $[M+Na]^+$, found m/z: 940.48837 $[M+H]^+$, 962.47032 $[M+Na]^+$.

1.3 Oligonucleotide synthesis

Synthesis of oligonucleotides HSc-2, HSc-9 and HSc-9.1(ON1-ON6) **(Table S1)** was performed in a 1 μmolar scale using the trityl-off mode. Each phosphoramidite was diluted to a 0.1 M solution and 0.3 M BTT solution in MeCN was used as an activator. Iodine solution (0.02 M) in THF/pyridine/water (ratio 70:20:10) was used for the oxidation step. Standard cycle procedures provided by BioAutomation Corporation were applied for the unmodified and modified phosphoramidites. The coupling volume and duration for the natural phosphoramidites were 220 μL and 1 minute 30 seconds whereas for the modified phosphoramidite it was increased to 300 μL and 6 minutes. Cleavage from the solid-phase was performed by 30%

aqueous NH_3 for 45 minutes (2×1 mL). Following deprotection step was carried out by incubation of the oligonucleotide solutions at 55 °C for 6 hours. The purification of the oligonucleotides was performed using HPLC with a linear gradient of MeCN (0–50%) in 0.1 M TEAB buffer (pH 7.6). The final lyofilization from H_2O provided pure products. The sequences of all chemically-synthesized oligonucleotides are shown in **Table S1**.

Supplementary Table S1 List of the chemically synthesized modified oligonucleotides ON1-ON6, the length (number of nucleotides), and number of modified 2′-deoxy-7-deazaadenosines (**dA***).

Code	Sequence (5′⟶3′)	Length (nt)	Number of **dA***
HSc-9.1<br>ON1	A*GA*CCTCA*TA*GTCCTTCTA*A*TTA*TA*CTCCA*<br>A*TGGCA*TA*GCTGGCA*C	46	13
HSc-9.1[a]<br>ON2	FAM-A*GA*CCTCA*TA*GTCCTTCTA*A*TTA*TA*CT<br>CCA*A*TGGC<br>A*TA*GC TGGCA*C	46	13
HSc-9<br>ON3	GCAGCAGAGATAGACGCTAA*GA*CCTCA*TA*GT<br>CCTTCTA*A*TTA*TA*CTCCA*A*TGGCA*TA*GCTG<br>GCA*C	65	13
HSc-9[a]<br>ON4	FAM-GCAGCAGAGATAGACGCTAA*GA*CCTCA*T<br>A*GTCTTCTA*A*TTA*TA*CTCCA*A*TGGCA*TA*GC<br>TGGCA*C	65	13
HSc-2<br>ON5	GCAGCAGAGATAGACGCTATTCTCTA*GA*CCTTC<br>TA*A*TTTA*CA*TA*CTCCA*A*TGGCA*TA*GCTGGC<br>A*C	65	12
HSc-2[a]<br>ON6	FAM-GCAGCAGAGATAGACGCTATTCTCTA*GA*C<br>CTTCTA*A*TTTA*CA*TA*CTCCA*A*TGGCA*TA*GC<br>TGGCA*C	65	12

[a] 5′-6-FAM-labelled

1.4 NMR Spectra

^{1}H,^{13}C NMR spectra of compound 2 (dA^{EEPh})

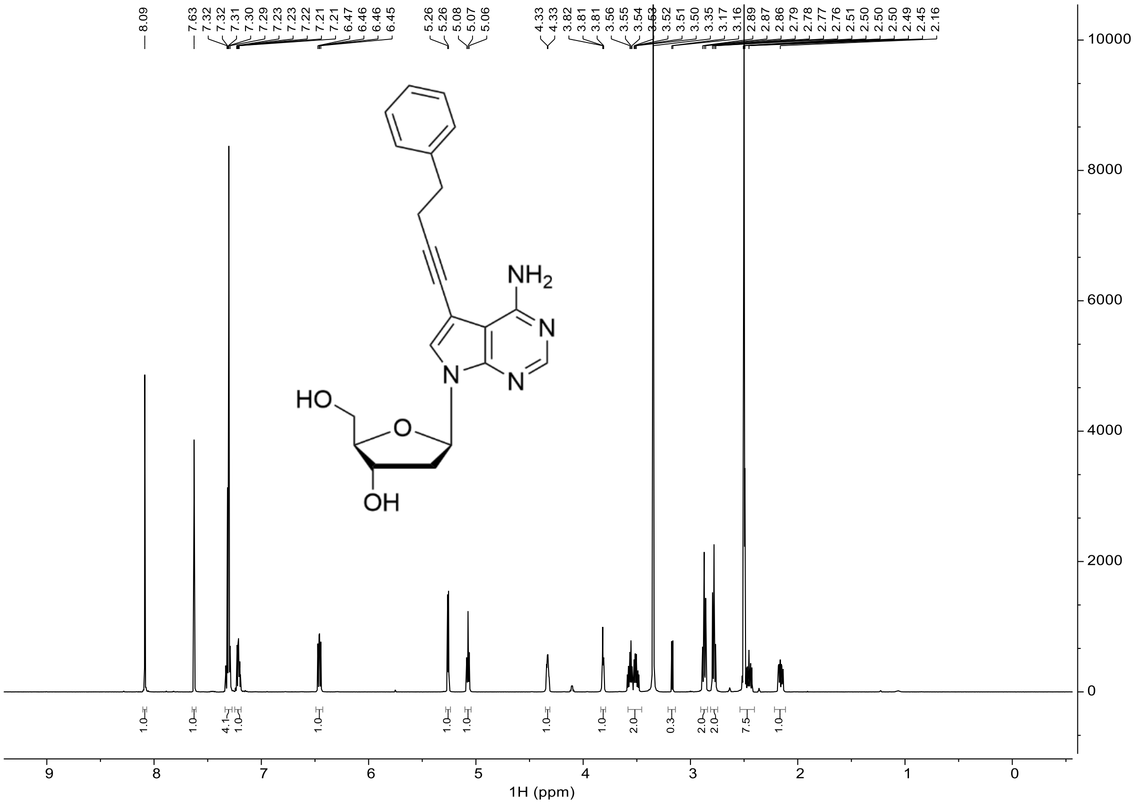

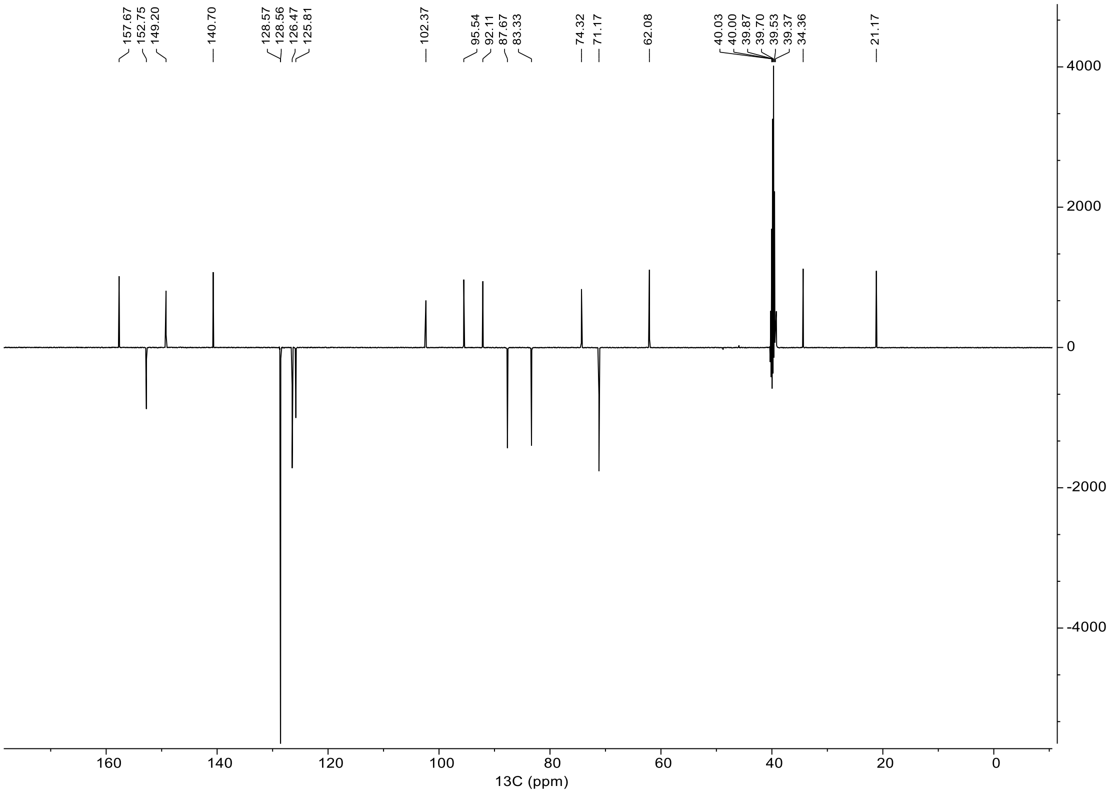

^{1}H,^{13}C NMR spectra of compound 3 (dA^{BuPh})

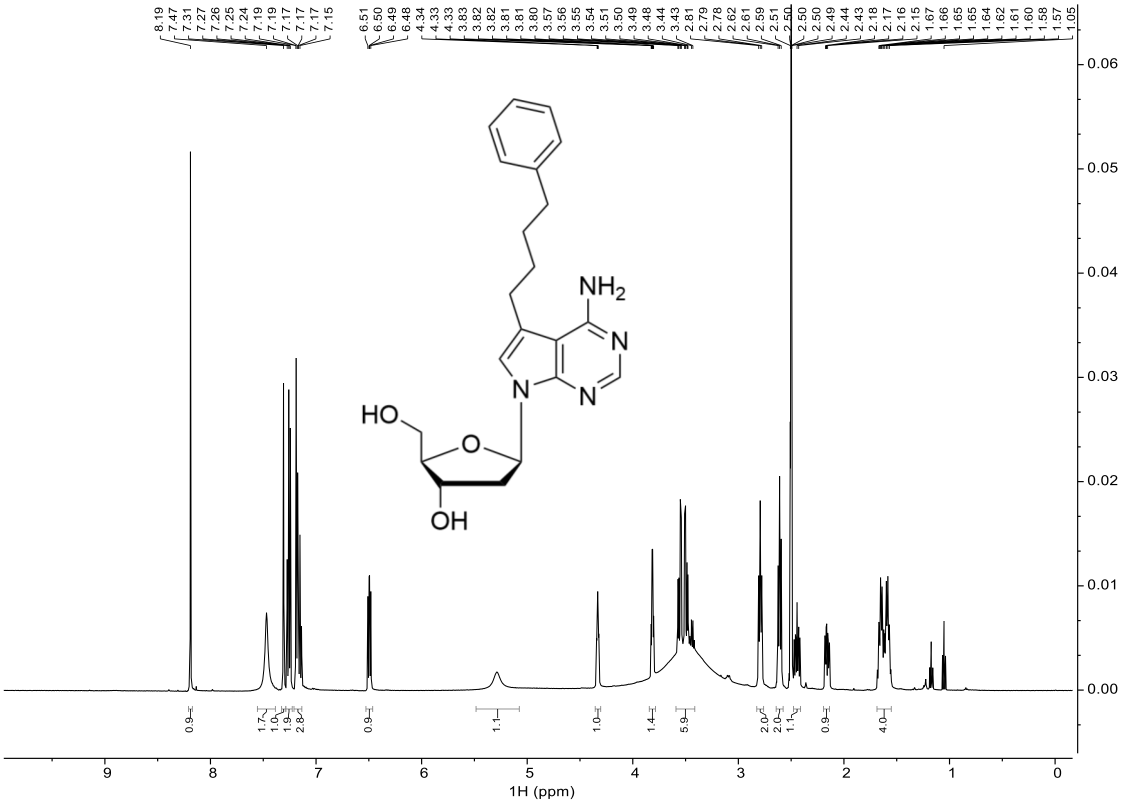

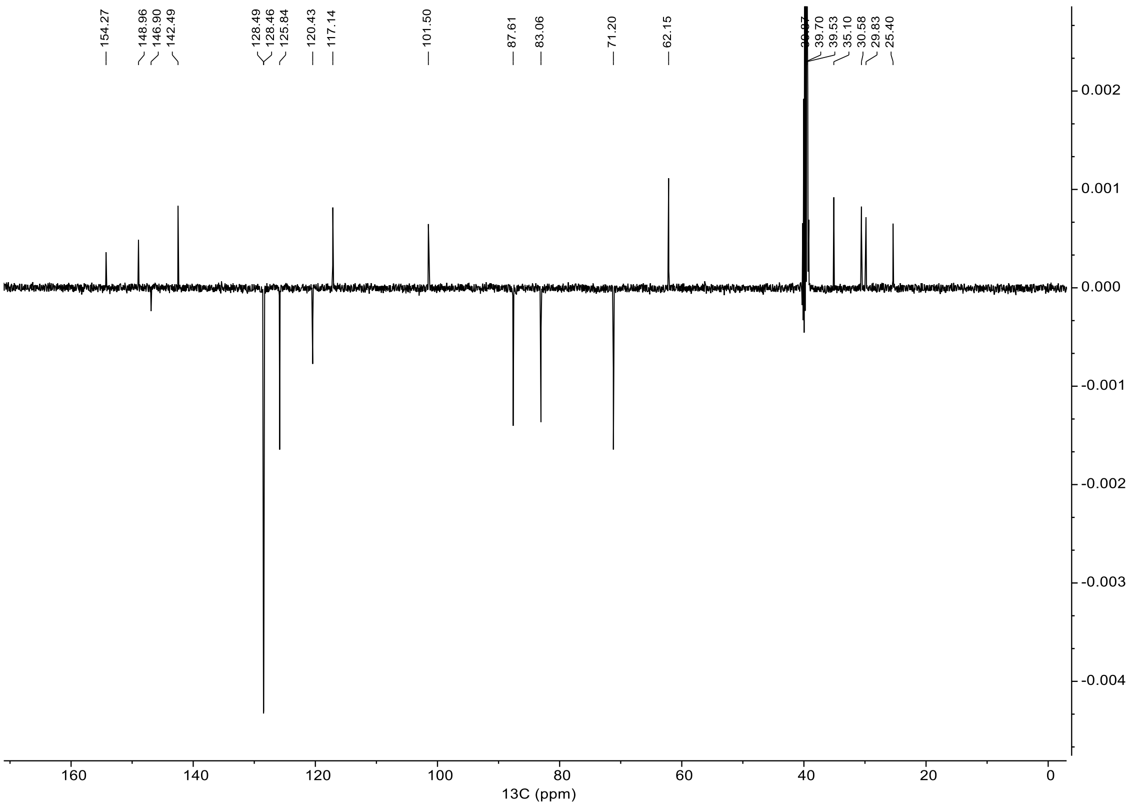

^{1}H, ^{13}C, $^{31}P\{^{1}H\}$ NMR spectra of compound 4 (dA^{BuPh}TP)

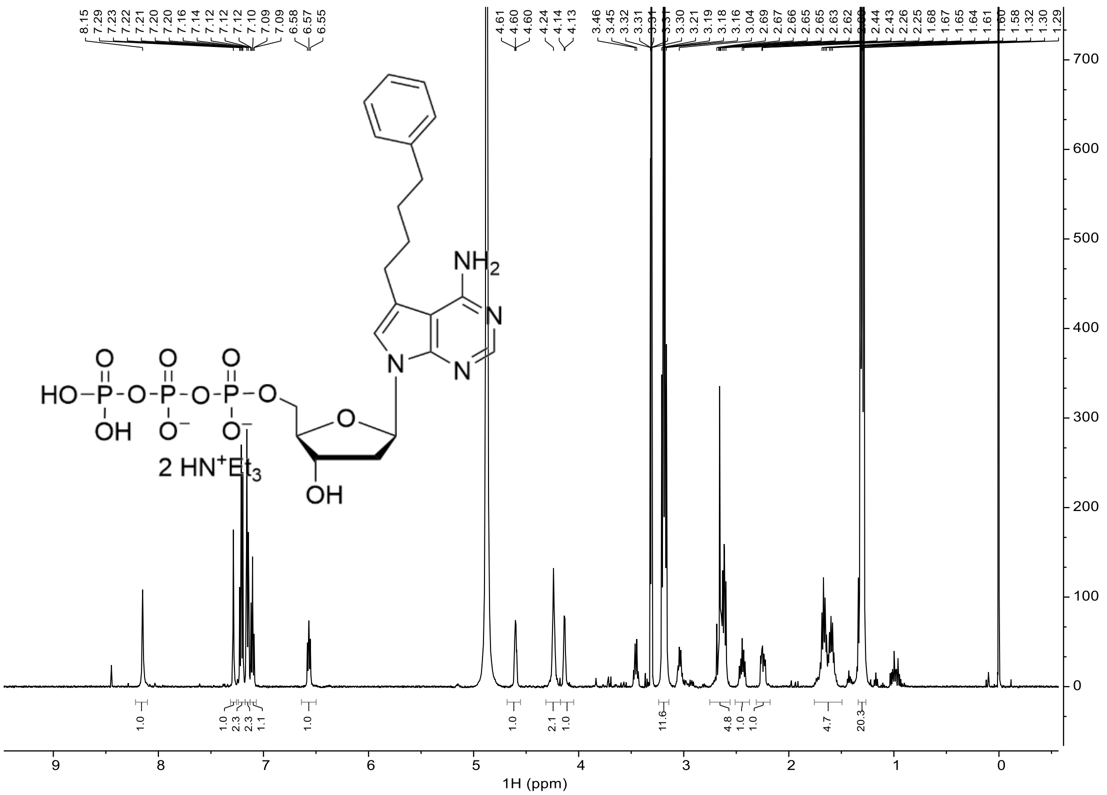

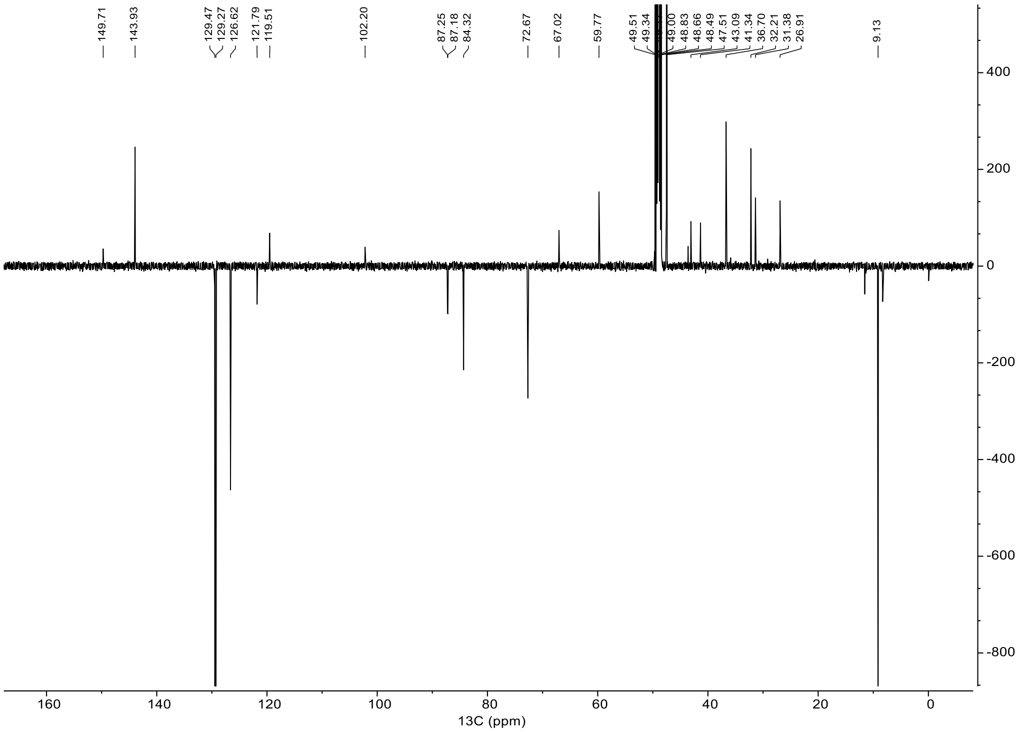

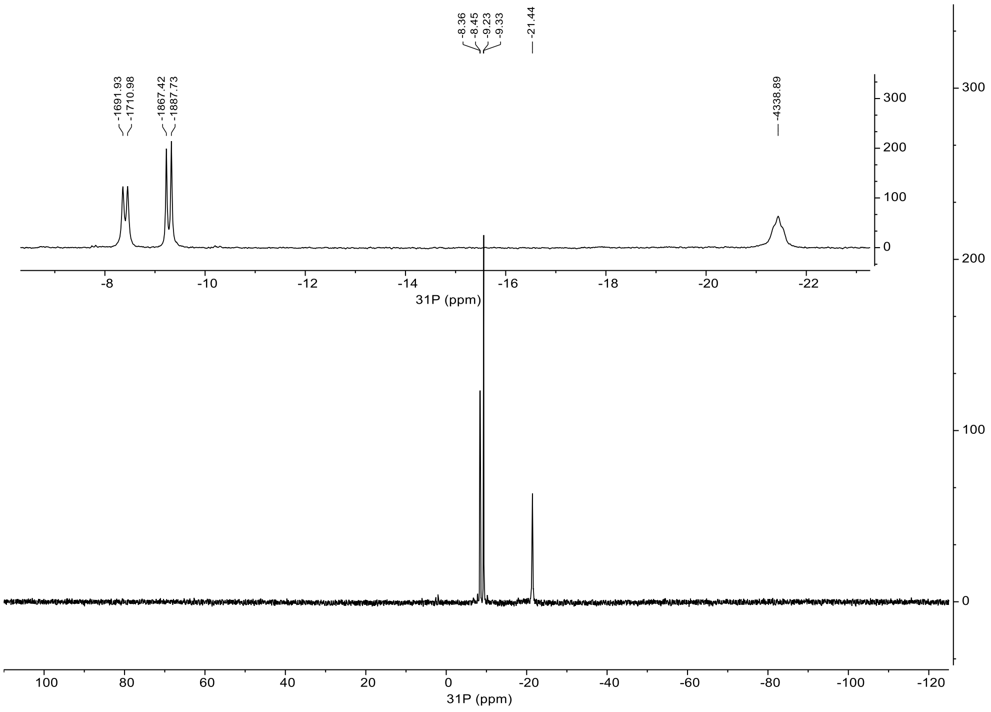

-8.36
-8.45
-9.23
-9.33
-21.44
-1691.93
-1710.98
-1867.42
-1887.73
-4338.89
31P (ppm)
31P (ppm)

^{1}H,^{13}C NMR spectra of compound 5

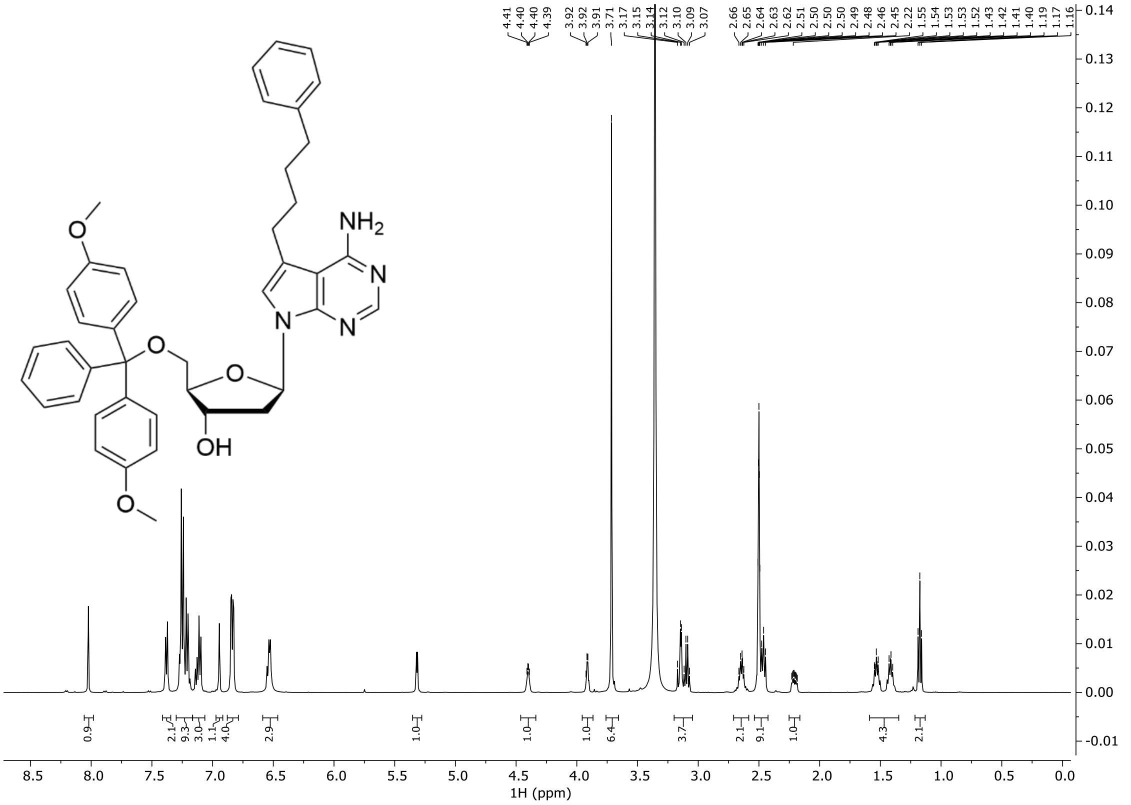

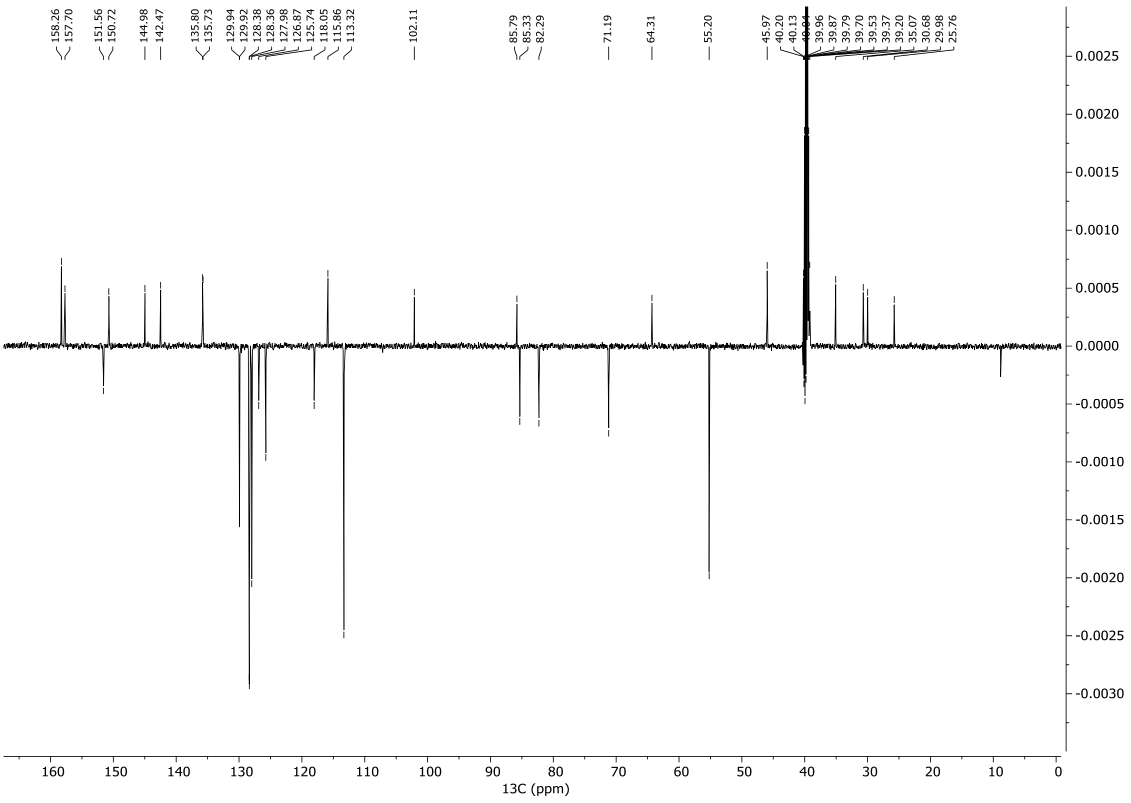

^{1}H,^{13}C NMR spectra of compound 6

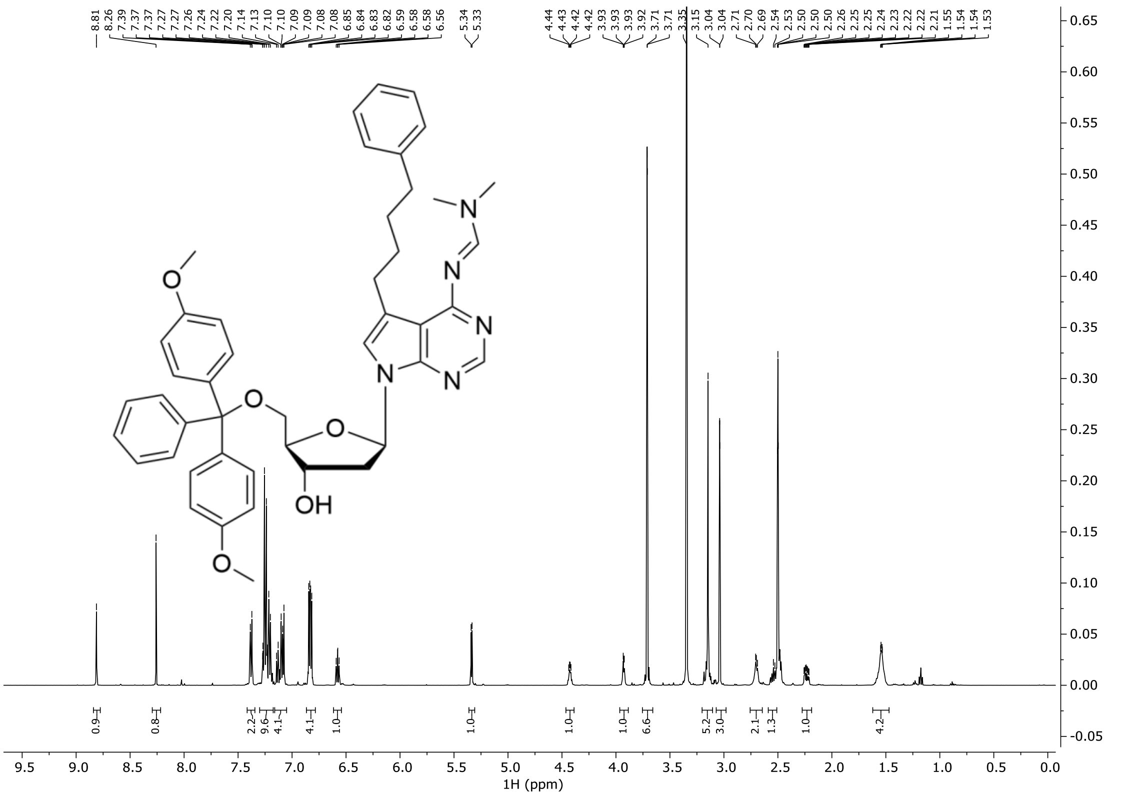

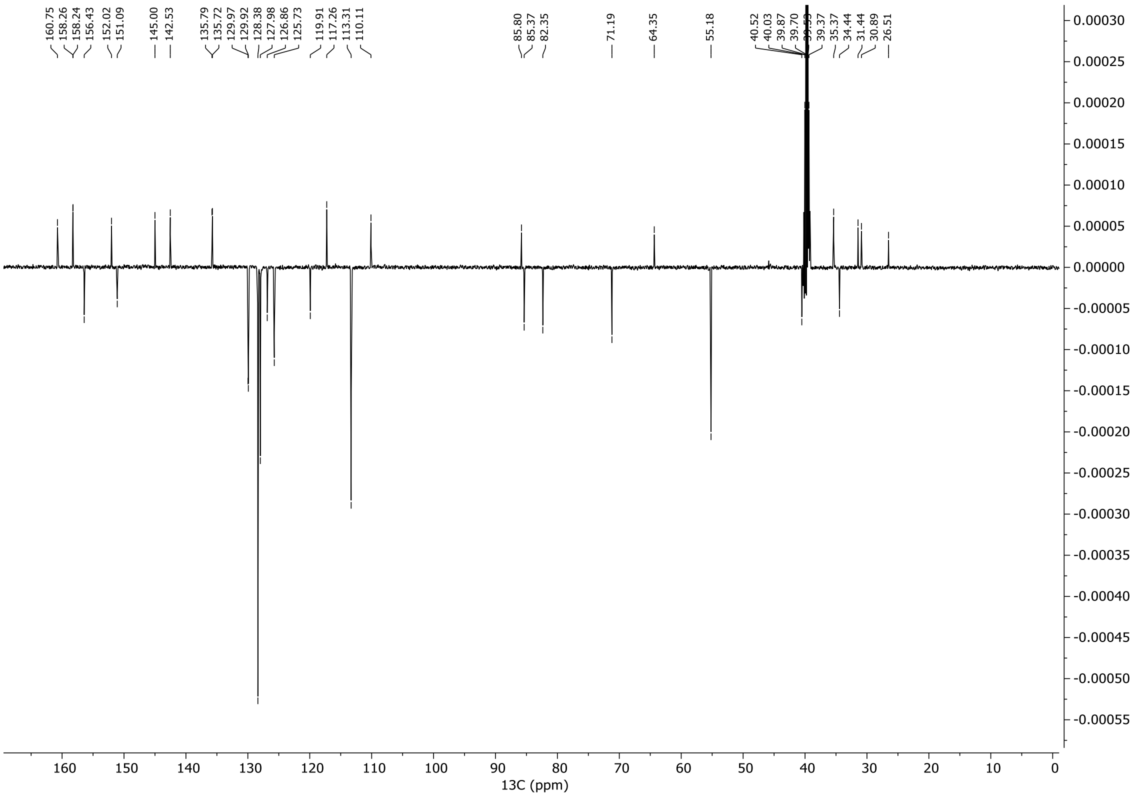

^{1}H, ^{13}C, ^{31}P NMR spectra of compound 7

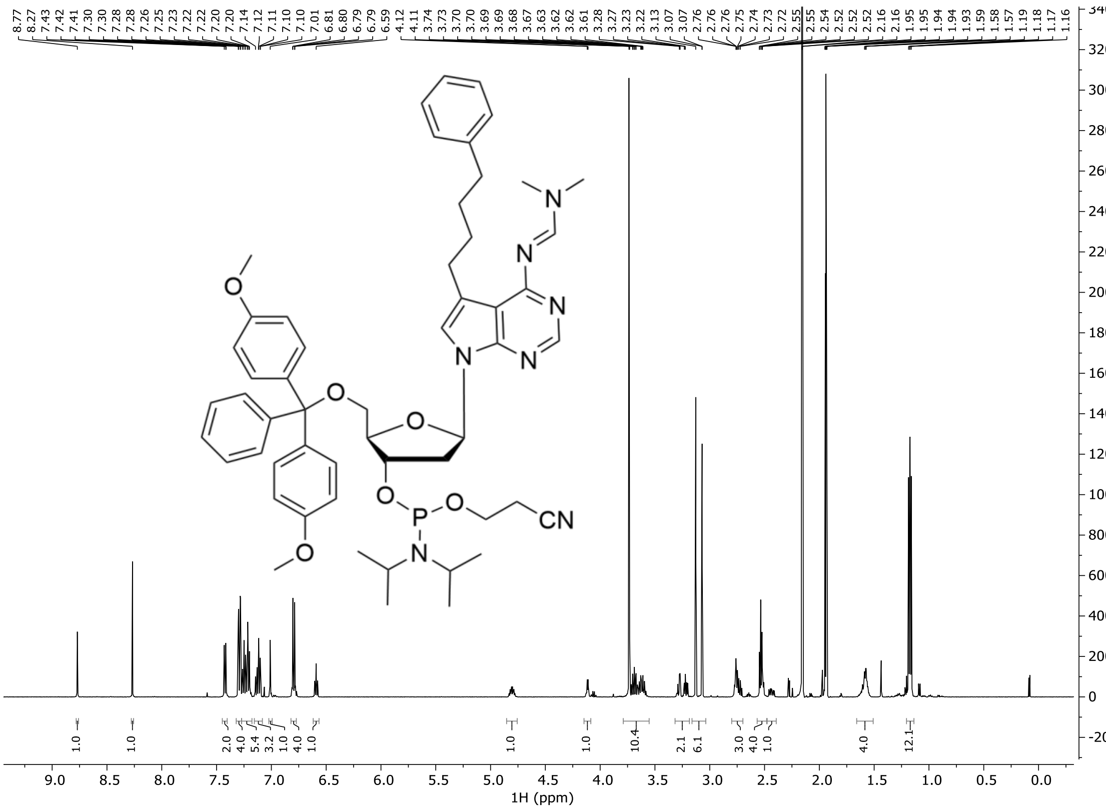

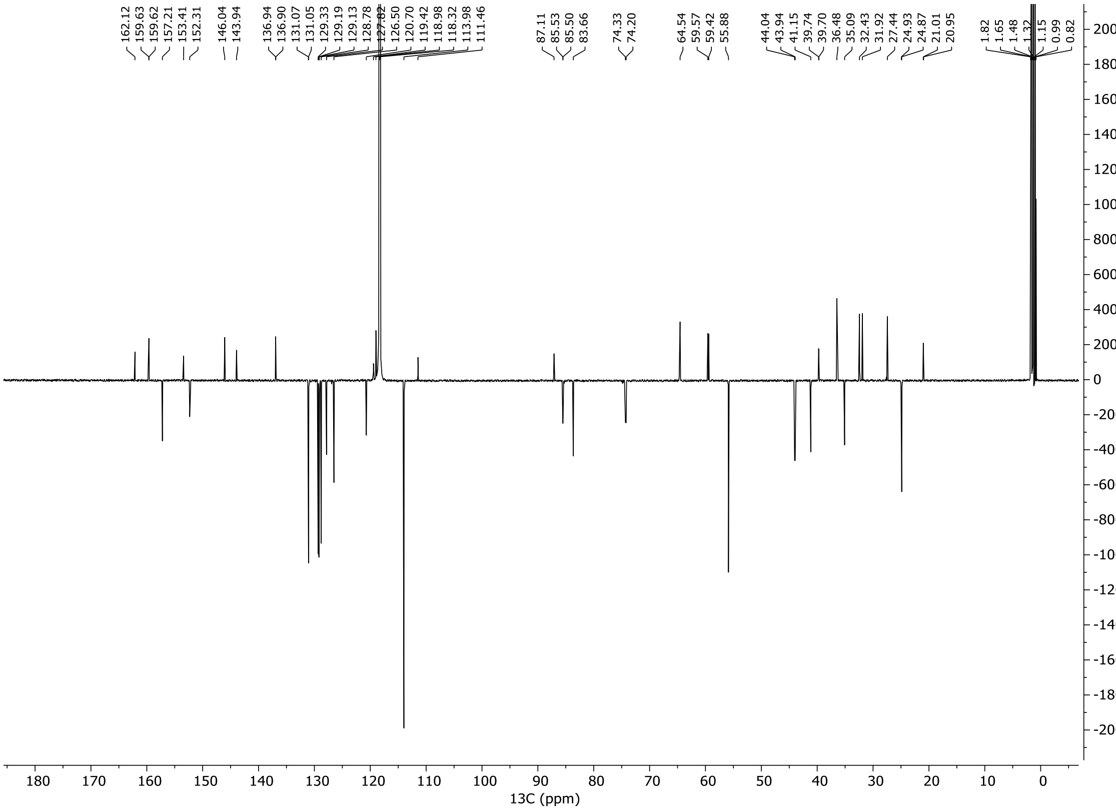

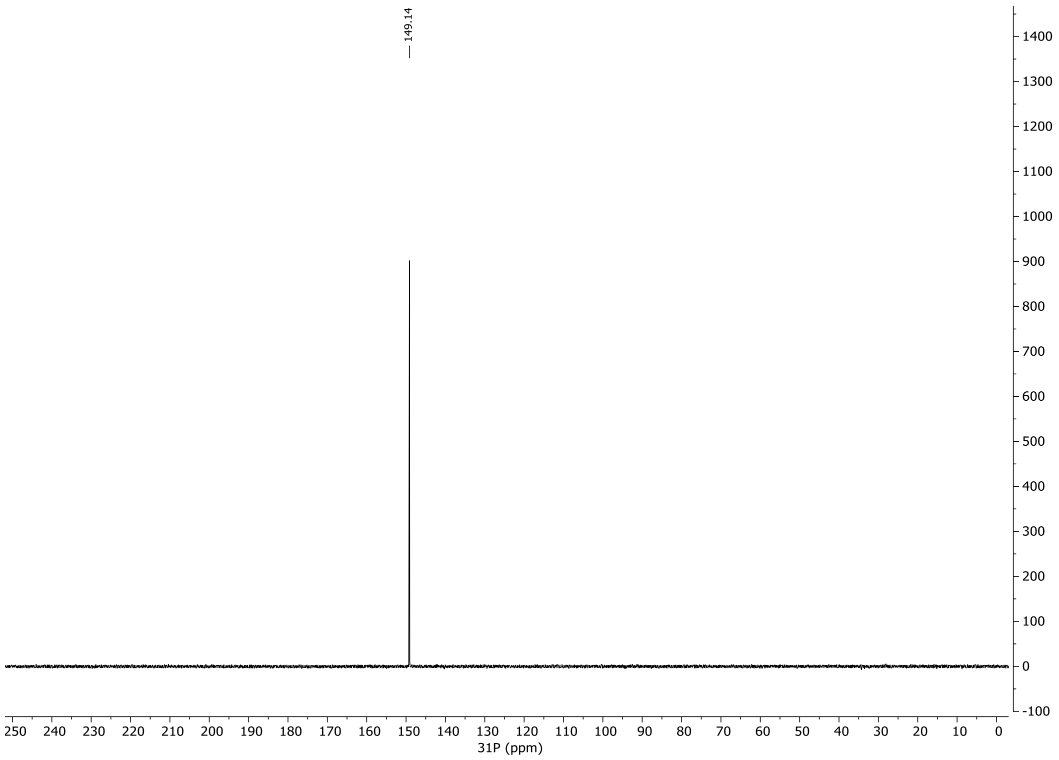

2

1.5 MALDI-TOF Mass Spectra

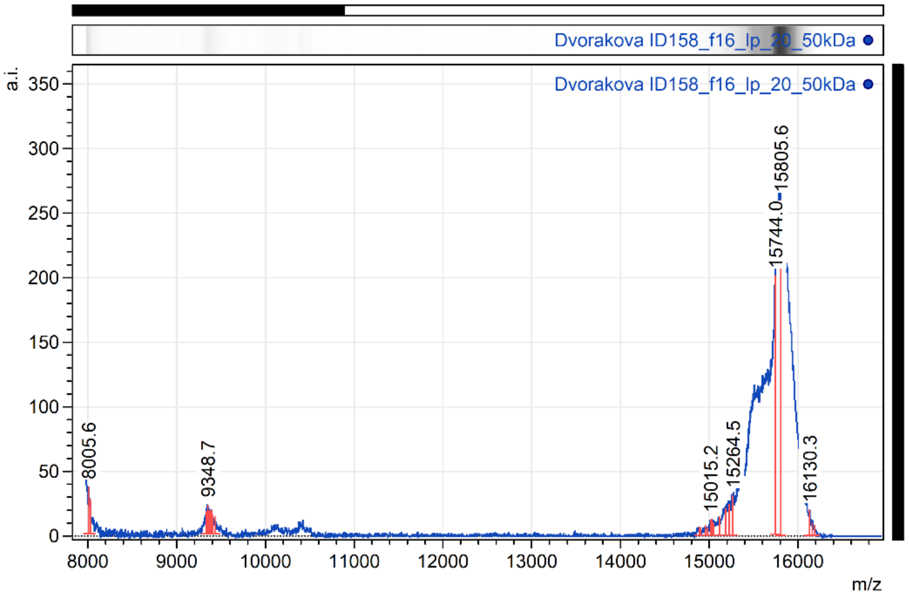

Figure S1. MALDI-TOF spectrum of **HSc-9.1**: calculated: 15745.5 Da; found: 15744.0 Da; Δ = 1.5 Da.

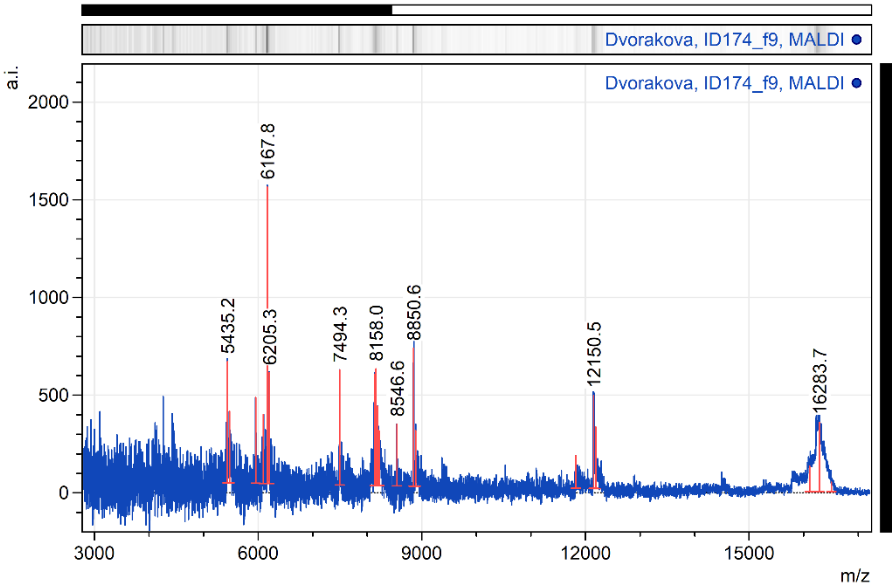

Figure S2. MALDI-TOF spectrum of **5’-6-FAM-labelled HSc-9.1**: calculated: 16283.5 Da; found: 16283.7 Da; Δ = 0.2 Da.

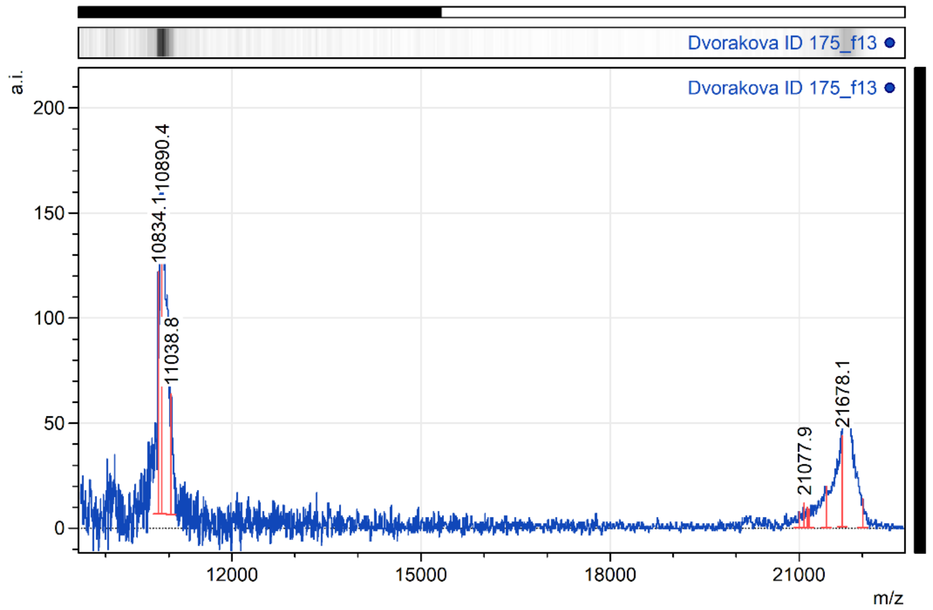

Figure S3. MALDI-TOF spectrum of **HSc-9**: calculated: 21678.3 Da; found: 21678.1 Da; Δ = 0.2 Da.

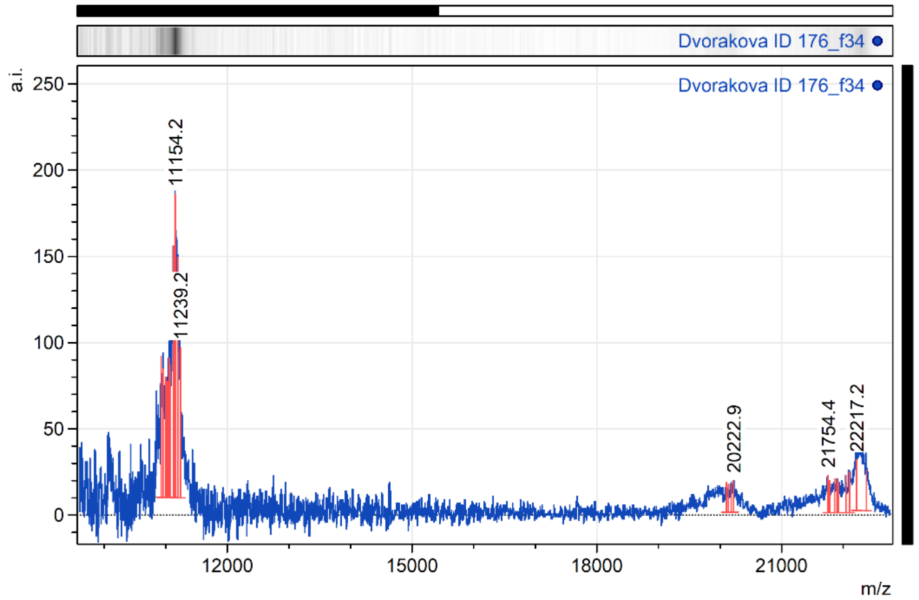

Figure S4. MALDI-TOF spectrum of **5'-6-FAM-labelled HSc-9**: calculated: 22216.3 Da; found: 22217.2 Da, Δ = 0.9 Da.

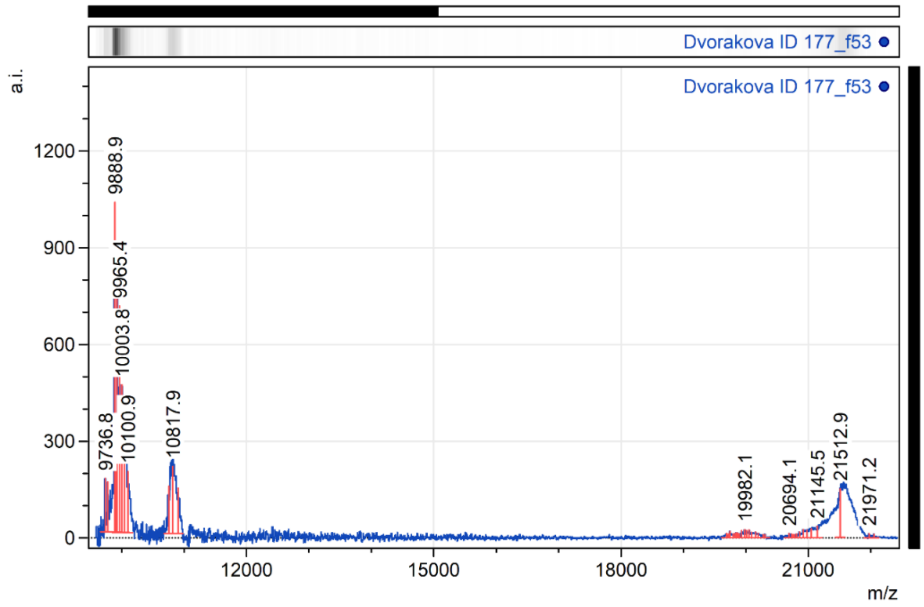

Figure S5. MALDI-TOF spectrum of **HSc-2**: calculated: 22512.2 Da; found: 22512.9 Da; Δ = 0.7 Da.

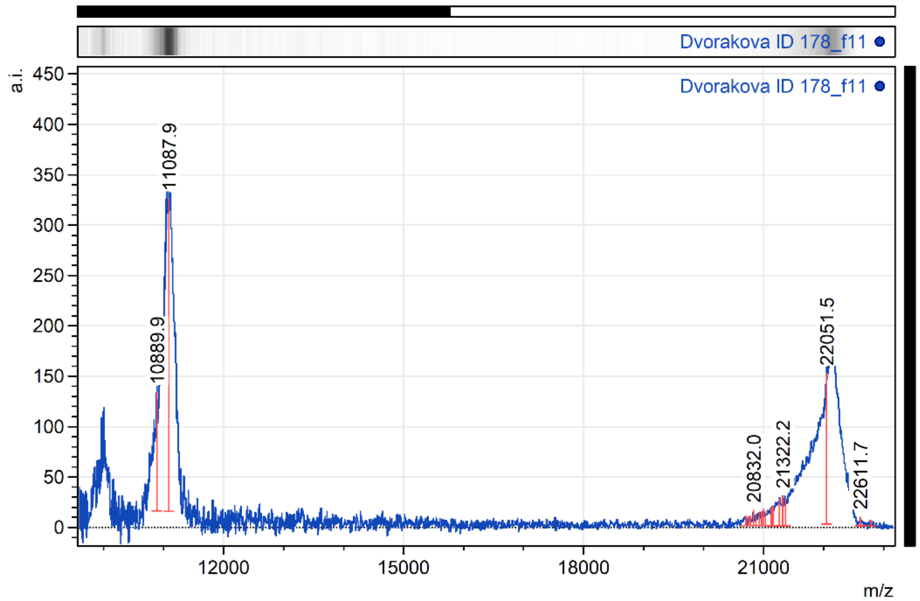

Figure S6. MALDI-TOF spectrum of **5'-6-FAM- labelled HSc-2**: calculated: 22050.2 Da; found: 22051.5 Da, Δ = 1.3 Da.

2. Experimental section – Biochemistry

2.1 General Remarks

All synthetic natural oligonucleotides (**Table 1**) were purchased from Generi Biotech (Czech Republic). Natural nucleoside triphosphates (dATP, dGTP, dTTP, dCTP) were purchased from either ThermoFisher Scientific or New England Biolabs. KOD XL and corresponding polymerase reaction buffers were purchased from Novagen. 5X colourless Go-Taq Flexi Buffer and Go Taq Flexi DNA polymerase obtained from Promega (USA). The PCR and PEX reaction were performed on a VWR Dopio gradient Thermal Cycler (ThermoScientific). PCR products were purified by Macherey-Nagel NucleoSpin gel and PCR clean-up system from ThermoScientific or QIAquick Nucleotide Removal Kit from Qiagen. HISPUR-Ni-NTA magnetic beads for the immobilisation of His-tagged Hsp70 were purchased from Invitrogen. HEPES was obtained from Fisher Bioreagents (Belgium), Bovine Serum Albumin (BSA), potassium chloride, magnesium chloride, sodium chloride, and other chemical reagents were obtained from Sigma-Aldrich. PAGE stop solution used after PEX reactions contained: 95% [v/v] formamide, 0.5 mM EDTA, 0.025% [w/v] bromophenol blue, 0.025%, [w/v] xylene cyanol FF, 0.025% [w/v] SDS in Milli-Q water. Samples after PEX reactions were always separated on a 12.5% PAGE (acrylamide/bisacrylamide 19:1 25% urea) under denaturing conditions in 1X TBE buffer (42 mA, 1 h) and were analyzed by fluorescence imaging using Typhoon FLA 9500 (GE Healthcare) Life Sciences. Samples after PCR reactions were always separated with a 3% agarose gel (Serva) in 0.5X TBE buffer (120 V, 2 h) using 6X DNA Gel Loading Dye (ThermoFisher Scientific). Ultra-low range DNA ladder (ThermoScientific) was used as a visual aid for band size. The visualization of PCR products was done by a combination of the intercalating agent GelRed (10,000X in water, Biotium, WVR International s.r.o (part of Avantor) and fluorescence imaging of 6-FAM label (6-carboxyfluorescein at 5´-end of ONs using Typhoon FLA 9500. PEX products were purified by Streptavidin Dynabeads myOne Streptavidin C1, which were purchased from Invitrogen. Bio layer interferometry (BLI) (OctetRED 96 system, ForteBio) using nickel coated biosensors (Octet NTA Biosensor, Sartorius) and plate-based binding assay using Pierce nickel coated black 96 well plates was used for the determination of binding affinity [K_d]. The BLI sensor chips were obtained from Forte Bio (Shanghai, China). Nickel coated black-96 well plates or streptavidin coated black-96 well plates were purchased from ThermoScientific (Czech Republic). All fluorescence intensity

measurements for the binding studies were measured with excitation at 480 nm and emission at 518 nm using Tecan microplate reader (Spark, Tecan). 3'-biotinylation of the modified aptamers was carried out using ddUTP-16 biotin (Jena Bioscience), 1X TdT buffer (New England Biolabs) and TdT NEB polymerase (New England Biolabs). 1X binding buffer consisted of 25mM HEPES, 5mM $MgCl_2$ and 300mM KCl and was prepared using Milli-Q ultrapure water. The anti-HSP70 antibody labelled with Alexa Fluor was obtained from Biolegend (USA).

2.2 **Cloning and protein purification of His-tagged Hsp70**

Coding sequences were cloned by Gateway recombinant technology (Invitrogen, Carlsbad, CA). Sequence encoding human HSP70 (HSPA1; NM_005345.6) was cloned into pDEST17 vector, containing an N-terminal polyhistidine-tag, and transformed into BL21 (DE3) RIPL cells (Agilent Technologies, Inc., Santa Clara, CA). Cells were grown in Luria-Bertani (LB) medium (37°C, 140 RPM) until an OD 600 of 0,5. Once the cell density was reached, temperature was reduced to 30°C and 1mM IPTG was added to induce an expression. After 4 hours incubation (30°C, 140 RPM), bacterial culture was pelleted by centrifugation. Cell pellet was resuspended in chilled lysis buffer (25mM TRIS, pH 7.5, 300mM KCl, 1 mg/ml lysozyme, 1mM PMSF) and additionally lysed by sonication. Bacterial lysate was obtained by centrifugation for 30 minutes at 10 000 g. 6xHis-Hsp70 protein was captured on HisTrap FF column (GE Healthcare) connected to ÄKTA pure L1 chromatography system (Cytiva). Unbound fraction was wash out using equilibration buffer (25mM TRIS, pH 7.5, 300mM KCl). Bound protein was eluted with equilibration buffer supplemented with 250mM Imidazole. Hsp70 fractions were concentrated, buffer exchanged to equilibration buffer (25mM TRIS, pH 7.5, 300mM KCl), flash frozen in liquid nitrogen and stored at -80°C for later use.

2.3 The use of modified dA*TP for incorporation into DNA by PEX

The PEX reaction mixture with a total volume of 20 µL for a single reaction contained: 31-mer template, prb4basII (sequence 5'-CTAGCATGAGCTCAGTCCCATGCCGCCCATG3'-) (100 µM, 2 µL), 5´-(6- FAM)-labelled 15-mer prim248short primer (CATGGGCGGCATGGG) (100 µM, 2.4 µL), natural dCTP, dGTP, dTTP (5 mM, 2 µL), modified **dA*TP** (5 mM, 2 µL), KOD XL DNA polymerase (2.5 U/µL, 1 µL) and KOD XL reaction buffer (10X, 2 µL). The PEX reaction mixture was incubated for 5 minutes at 95 °C, followed by 1.5 minutes at 55 °C and then followed by 30 min at 60 °C. The reaction was stopped by addition of PAGE stop solution (20 µL) and denatured for 3 minutes at 95 °C. Samples were analyzed by PAGE and visualized using fluorescence imaging (**Figure S7**).

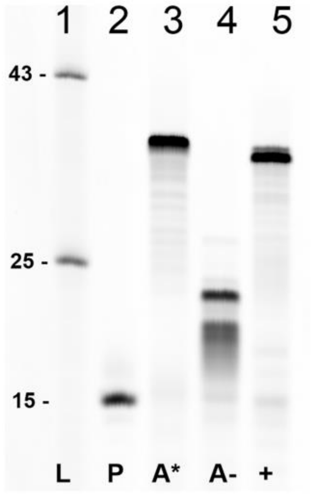

Figure S7. Denaturing PAGE analysis of PEX reaction with modified **dA*TP** and 31-mer template: Lane 1 (L): single-stranded DNA ladder, lane 2 (P): 6-FAM-labelled primer, lane 3 (A*): **dA*TP** + dCTP, dGTP, dTTP, lane 4 (A-): no dATP added, lane 5 (+): natural dNTPs.

2.4 Example of gel electrophoresis of amplified 65 bp product (from round 1)

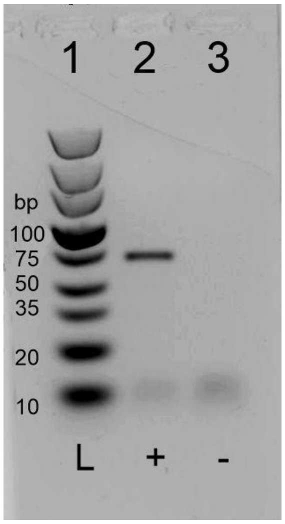

Figure S8. 3% Agarose gel analysis of PCR reaction after amplification of ssDNA from round 1 of SELEX using Go-Taq DNA polymerase and natural dNTPs. Lane 1 (L): double-stranded DNA ladder, lane 2 (+): amplified product after 12 PCR cycles from round 1, lane 3 (-): negative control, PCR mix without template.

2.5 Example of gel electrophoresis of ssDNA generation (from round 1)

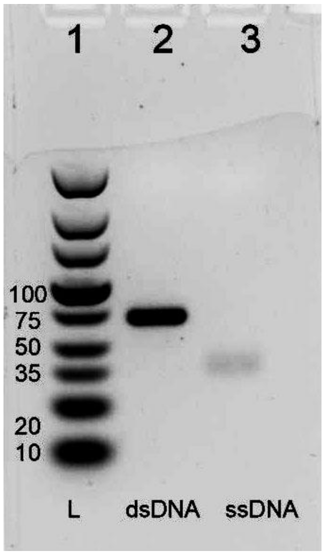

Figure S9. 3% Agarose gel analysis of Lambda exonuclease digestion from dsPCR product from round 1. Lane 1 (L): double-stranded DNA ladder: lane 2 (dsDNA): amplified product after 12 PCR cycles from round 1, lane 3 (ssDNA): ssDNA product produced by the Lambda exonuclease (the product migrates faster in the gel and appears as a lower band indicating successful ssDNA generation).

2.6 Identification of aptamer sequences

After 9 rounds of selection the enriched pools of DNA were prepared for testing by Next-generation sequencing (NGS). In order to prepare the DNA for sequencing, PCR of rounds 0-9 was performed in-order to introduce the adapter followed by the index sequences **(Table 1).** For the addition of the adapter sequences, the PCR mixture with a total volume of 25 µL contained: ssDNA (50 nM, 2 µL) from each round, P-For$^{OvH+For}$ and P-Rev$^{OvH+Rev}$ (10 µM, 0.75 µL, each) and 2X KAPA HiFi Hotstart Master Mix (12.5 µL) as supplied by the manufacturer. The PCR reaction was performed under the following cycling conditions: 95 °C for 3 min, followed by 35 cycles at 98 °C for 20 sec, 64 °C for 15 sec, and 72 °C for 15 sec, followed by a final elongation step at 72 °C for 1 min. For PCR with index primers, the PCR mixture with a total volume of 25 µL contained: purified dsDNA from adapter PCR step (5 µL used directly), P-For^{Ind} and P-Rev^{Ind} (10 µM, 2.5 µL, each) and 2X KAPA HiFi Hotstart Master Mix (12.5 µL) as supplied by the manufacturer. The PCR reaction was performed under the following cycling conditions: 95 °C for 3 min, followed by 8 cycles at 98 °C for 20 sec, 64 °C for 15 sec, and 72 °C for 15 sec, followed by a final elongation step at 72 °C for 1 min. The purified pools were quantified by qPCR, normalized to a final 10 nM concentration and sequenced using Nextera XT 2 Mid-Output cartridge and MiniSeq sequencing system (Illumina).

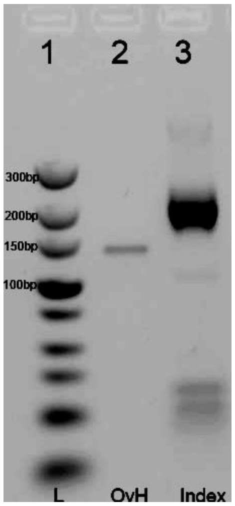

Figure S10. 2% Agarose gel analysis of PCR product obtained by adapter and index PCR using the 2X KAPA HiFi Hotstart Master Mix: Lane 1 (L): double-stranded DNA ladder; Lane 2

(OvH): 150 bp product obtained after PCR with overhang adapter primers; Lane 3 (Index): 230 bp product obtained after PCR with index primers.

2.7 Enzymatic synthesis and initial screening of candidates HSe-1 to HSe-9

The PEX reaction mixture with a total volume of 20 µL for a single reaction contained: reverse complement sequences of 5'-biotinylated Hsp70 aptamers 1-9 (**Table 3, main manuscript**) (100 µM, 2 µL), 5´-(6- FAM)-labelled primer Rev$^{Lib\ c}$ (**Table 1, main manuscript**) (100 µM, 2.4 µL), natural dCTP, dGTP, dTTP (5 mM, 2 µL), modified **dA*TP** (5 mM, 2 µL), KOD XL DNA polymerase (2.5 U/µL, 1 µL) and KOD XL reaction buffer (10X, 2 µL). The PEX reaction mixture was incubated for 5 minutes at 95 °C, followed by 1.5 minutes at 55 °C and then followed by 2 hours at 60 °C. The reaction was stopped by addition of PAGE stop solution (20 µL) and denatured for 3 minutes at 95 °C. The subsequent ssDNA generation using C1 Dynabeads and ssDNA release, was carried out as described in the main manuscript. Samples were analyzed by PAGE and visualized using fluorescence imaging (**Figure S11**). The enzymatically synthesized modified aptamers were assigned as HSe-1 to HSe-9.

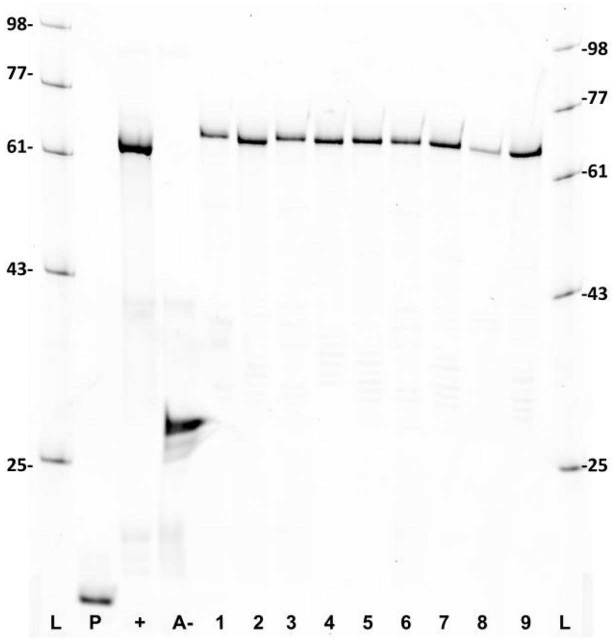

Figure S11. Denaturing PAGE analysis of PEX reaction with modified **dA*TP** and aptamers HSe-1 to HSe-9: (L): single-stranded DNA ladder, (P): 6-FAM labelled primer, (+): natural dNTPs, (A-): no dATP added, (1-9): enzymatically synthesized HSe-1 to HSe-9 aptamers using **dA*TP** + dCTP, dGTP, dTTP.

The initial screening of aptamers HSe-1 to HSe-9 was measured by a plate-based binding assay. The assay was performed in black nickel coated 96-well plates as follows: wells were washed 3 times with 200 µL of wash buffer (HEPES, 5 mM $MgCl_2$, 300 mM KCl, 1% BSA and 0.05% Tween 20). 100 µg of His-tagged Hsp70 in 100 µL of 1X binding buffer (25 mM HEPES, 5 mM $MgCl_2$, 300 mM KCl) was incubated in the wells for 1 hour at 24 °C with shaking at 300 rpm. As a negative control, wells containing 1X binding buffer only and without Hsp70 were included for background subtraction. All wells were washed with wash buffer and blocked with 200 µL of 3% BSA for 1 hour at 24 °C with shaking at 300 rpm. 5'-6-FAM-labelled aptamer candidates were prepared enzymatically as described above, and 100 µL of each aptamer at 300 nM was heated to 95 °C, cooled for 30 minutes at room temperature to allow folding, and added to wells containing Hsp70 and also control wells without Hsp70 for 1 hour at 24 °C with shaking at 300 rpm. As a positive control, 100 µL containing 5 µg of anti-Hsp70 antibody (labelled with Alexa fluor) was added to wells with and without Hsp70 and incubated for 1 hour in the dark at 24 °C with shaking at 300 rpm. Wells were then washed 5 times with 200 µL of wash buffer and finally 100 µL of 1X binding buffer was added before the measurement. Fluorescence intensity was measured using Tecan microplate reader as described in biochemistry general remarks.

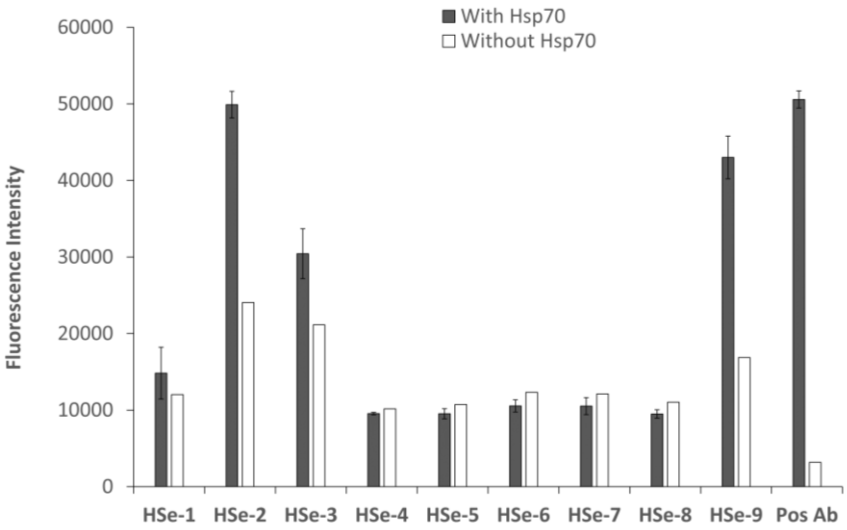

Figure S12. Aptamer screening results for candidates HSe-1 to HSe-9. Screening results obtained from a plate-based binding assay after His-tagged Hsp70 immobilisation to nickel coated black plates and incubation with 5'-6-FAM-labelled BuPh-modified **dA*TP** aptamers.

The positive control comes from Alexa fluor labelled anti-Hsp70 antibody. Background fluorescence for each aptamer without Hsp70 is included for comparison. Error bars are the mean ± SD of duplicate experiments.

2.8 In-house chemical synthesis of aptamers HSc-2 and HSc-9

Synthesis of oligonucleotides HSc-2, HSc-9 and HSc-9.1 **Supplementary Table S1** was performed in a 1 μmolar scale using the trityl-off mode. Each phosphoramidite was diluted to a 0.1 M solution and 0.3 M BTT solution in MeCN was used as an activator. Iodine solution (0.02 M) in THF/pyridine/water (ratio 70:20:10) was used for the oxidation step. Standard cycle procedures provided by BioAutomation Corporation were applied for the unmodified and modified phosphoramidites. The coupling volume and duration for the natural phosphoramidites were 220 μL and 1 minute 30 seconds whereas for the modified phosphoramidite it was increased to 300 μL and 6 minutes. Cleavage from the solid-phase was performed by 30% aqueous NH_3 for 45 minutes (2×1 mL). Following deprotection step was carried out by incubation of the oligonucleotide solutions at 55 °C for 6 hours. The purificati
on of the oligonucleotides was performed using HPLC with a linear gradient of MeCN (0–50%) in 0.1 M TEAB buffer (pH 7.6). The final lyophilization from H_2O provided pure products. The sequences of all chemically-synthesized oligonucleotides are shown in full detail in **Supplementary Table S1**.

2.9 BLI steady state raw data files for modified aptamer HSc-2

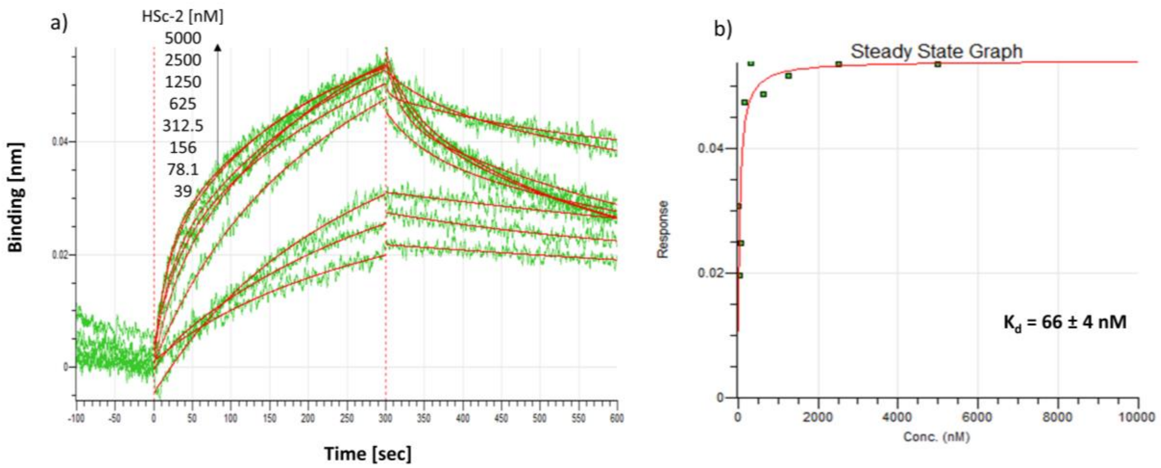

Figure S13. Bio-layer interferometry (BLI) used for determination of binding affinity. His-tagged Hsp70 (50 nM, 200 µL) immobilized onto nickel coated biosensors and tested with various concentrations of HSc-2 (39 nM to 5000 nM): (a) Processed kinetic data from the BLI showing the association (0-300 sec) and dissociation (300-600 sec) of the aptamer HSc-2, the overlaid fit and binding affinity value are also shown; (b) The steady state binding curve of HSc-2 to Hsp70.

2.10 **BLI steady state raw data files for natural aptamer HSNat-2**

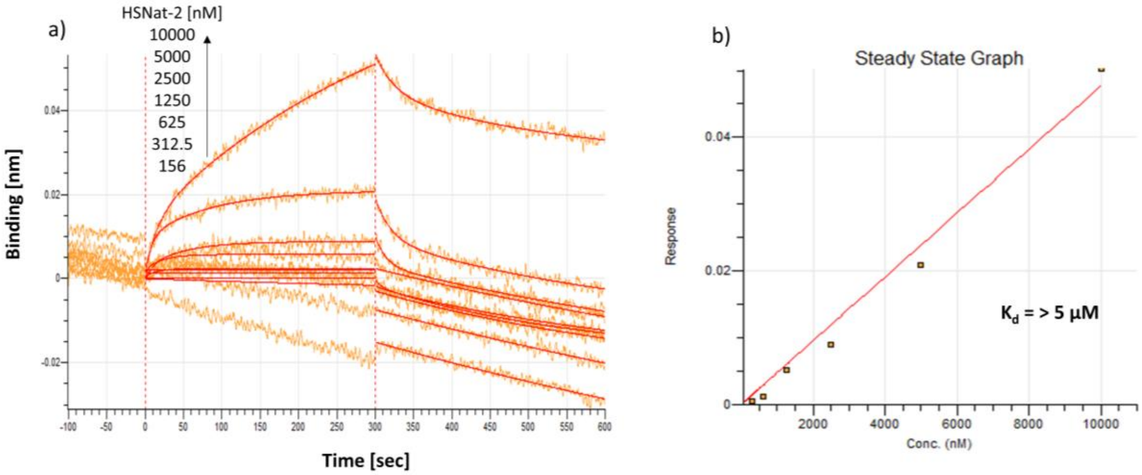

Figure S14. Bio-layer interferometry (BLI) used for determination of binding affinity. His-tagged Hsp70 (50 nM, 200 µL) immobilised onto nickel biosensors and tested with various concentrations of HSNat-2 (156 nM to 10000 nM): (a) Processed kinetic data from the BLI showing the association (0-300 sec) and dissociation (300-600 sec) of the aptamer HSNat-2, the overlaid fit and binding affinity value are also shown; (b) The steady state binding curve of HSNat-2 to Hsp70.

2.11 **BLI steady state raw data files for modified aptamer HSc-9**

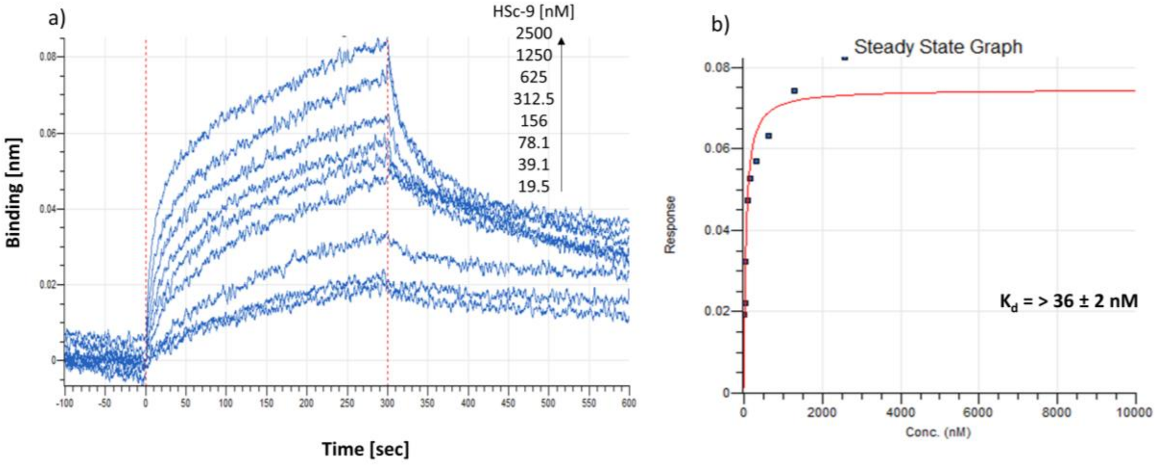

Figure S15. Bio-layer interferometry (BLI) used for determination of binding affinity. His-tagged Hsp70 (50 nM, 200 µL) immobilised onto nickel biosensors and tested with various concentrations of HSc-9 (19.5 nM to 2500 nM): (a) Processed kinetic data from the BLI showing the association (0-300 sec) and dissociation (300-600 sec) of the aptamer HSc-9, the overlaid fit and binding affinity value are also shown; (b) The steady state binding curve of HSc-9 to Hsp70.

2.12 **BLI steady state raw data files for natural aptamer HSNat-9**

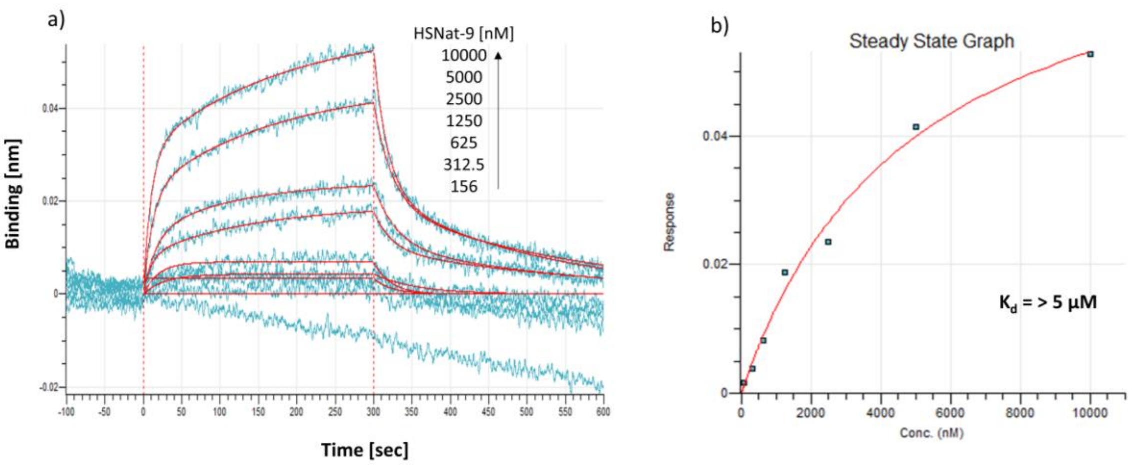

Figure S16. Bio-layer interferometry (BLI) used for determination of binding affinity. His-tagged Hsp70 (50 nM, 200 µL) immobilised onto nickel biosensors and tested with various concentrations of HSNat-9 (156 nM to 10000 nM): (a) Processed kinetic data from the BLI showing the association (0-300 sec) and dissociation (300-600 sec) of the aptamer HSNat-9, the overlaid fit and binding affinity value are also shown; (b) The steady state binding curve of HSNat-9 to Hsp70.

2.13 **BLI steady state raw data files for modified aptamer HSc-9.1**

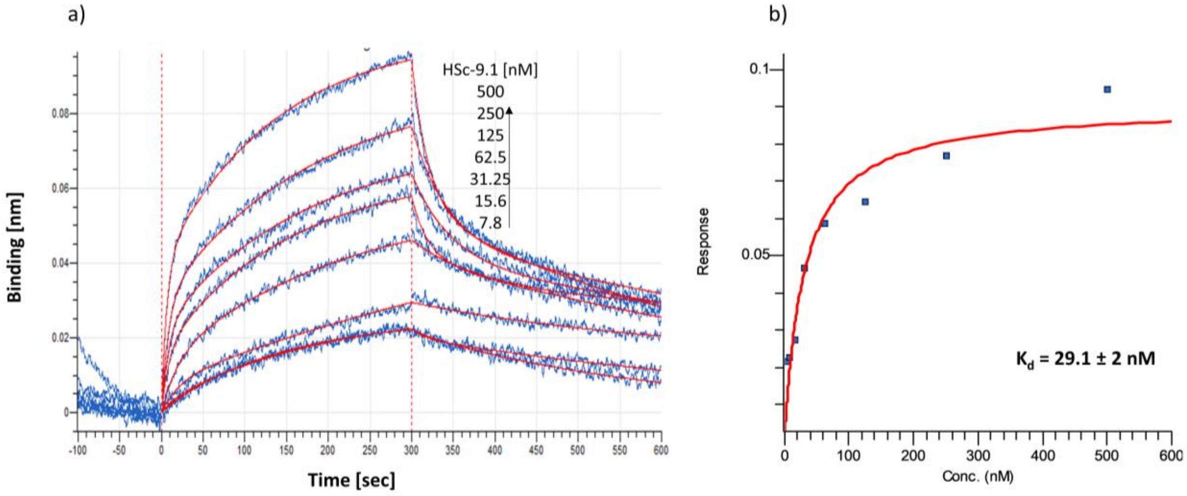

Figure S17. Bio-layer interferometry (BLI) used for determination of binding affinity. His-tagged Hsp70 (50 nM, 200 µL) immobilised onto nickel biosensors and tested with various concentrations of truncated aptamer HSc-9.1 (7.8 nM to 500 nM): (a) Processed kinetic data from the BLI showing the association (0-300 sec) and dissociation (300-600 sec) of the truncated aptamer HSc-9.1, the overlaid fit and binding affinity value are also shown; (b) The steady state binding curve of HSc-9.1 to Hsp70.

2.14 3'-Biotinylated HSc-9.1 as a capture aptamer for Hsp70 detection by anti-Hsp70 antibody in a sandwich-based ELISA

HSc-9.1 was first 3'-biotinylated using ddUTP-16 biotin and TdT polymerase to allow for the immobilisation onto streptavidin coated well plates. To produce the 3'-biotinylated HSc-9.1 aptamer, the reaction mixture with a total volume of 50 µL for a single reaction contained: HSc-9.1 (final concentration 3 µM, 32.5 µL each), ddUTP-16-biotin (final concentration 50 µM, 2.5 µL) TdT polymerase (2 U/ µL, 5 µL) and 1X TdT buffer (5 µL) and $CoCl_2$ (final concentration 0.25 mM, 5 µL). The reaction was performed at 37 °C for 1 hour followed by termination of the reaction at 70 °C for 10 min. The DNA was purified using QIAquick Nucleotide Removal Kit according to the manufactures' instructions and eluted in 45 µL of nuclease free water, followed by addition of 5 µL of 10X binding buffer (250 mM HEPES, 50 mM $MgCl_2$, 3000 mM KCl). The samples were quantified by nanodrop spectrophotometer, prepared at 50 nM concentration and were heated to 95 °C for 5 min and cooled to room temperature for 30 min to allow for aptamer folding. The ELISA assay was performed in black streptavidin coated 96-well plates as follows: wells were washed 3 times with 200 µL of wash buffer (HEPES, 5 mM $MgCl_2$, 300 mM KCl, 1% BSA and 0.05% Tween 20). Pre-folded 3'-biotinylated aptamer HSc-9.1 in 1X binding buffer (50 nM, 100 µL) was incubated in the wells for 2 hours at 24 °C with shaking at 300 rpm. The wells without aptamer were incubated with 100 µL of 1X binding buffer only under the same conditions. Wells were washed 3 times with 200 µL of wash buffer followed by incubation with either 100 µg of Hsp70 in 100 µL of 1X binding buffer or 100 µL of 1X binding buffer only. The reaction was incubated in the wells for 1 hour at 24 °C with shaking at 300 rpm. Wells were washed 3 times with 200 µL of wash buffer followed by incubation with either 100 µL containing 5 µg of anti-Hsp70 antibody (labelled with Alexa fluor) or 100 µL of 1X binding buffer only. The reaction was incubated for 1 hour in the dark at 24 °C with shaking at 300 rpm. Wells were then washed 5 times with 200 µL of wash buffer and finally 100 µL of 1X binding buffer was added before the measurement. Fluorescence intensity was measured using Tecan microplate reader as described in biochemistry general remarks.

Supplementary Table S2. List of control samples included in the initial sandwich ELISA

Sample	Sample description	Control type
1	HSc-9.1 in BB + Hsp70 + AB	Positive
2	HSc-9.1 in BB + AB	Negative
3	HSc9.1 in BB only	Negative
4	BB + Hsp70 + AB	Negative
5	BB only	Negative

Note: BB = binding buffer (1X); AB = Anti-Hsp70 antibody labelled with Alexa-flour

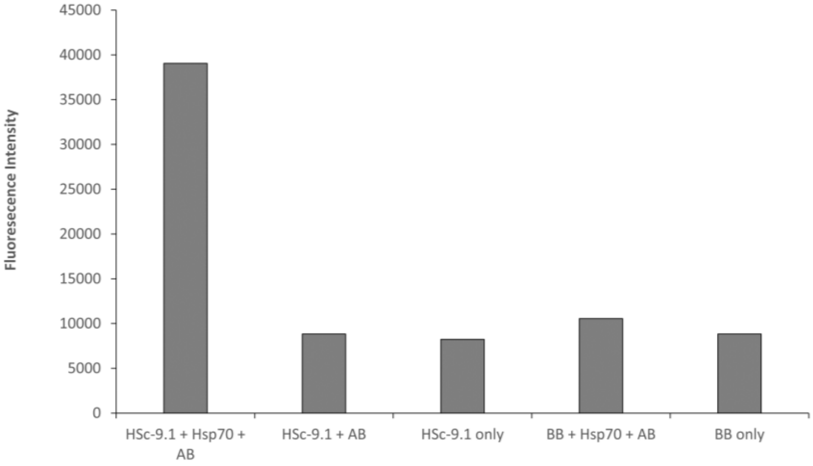

Figure S18. Validation of 3'-biotinylated HSc-9.1 as a capture agent for use in a sandwich ELISA including various positive and negative controls.

Note: BB = binding buffer (1X); AB = Anti-Hsp70 antibody labelled with Alexa-flour

For the sandwich ELISA and testing of the capture ability of the HSc-9.1 3’-biotinylated aptamer, the procedure was carried out in the same manner as described above. After immobilisation of the aptamer. The wells were washed 3 times with 200 µL of wash buffer followed by incubation with ten-fold serial dilution of Hsp70 (1000 µg to 0.1 µg in 100 µL of 1X binding buffer). The reaction was incubated in the wells for 1 hour at 24 °C with shaking at 300 rpm. Wells were washed 3 times with 200 µL of wash buffer followed by incubation with 100 µL containing 5 µg of anti-Hsp70 antibody (labelled with Alexa fluor) or 100 µL of 1X binding buffer only. The reaction was incubated for 1 hour in the dark at 24 °C with shaking at 300 rpm. Wells were then washed 5 times with 200 µL of wash buffer and finally 100 µL of 1X binding buffer was added before the measurement. Fluorescence intensity was measured using Tecan microplate reader as described in biochemistry general remarks.

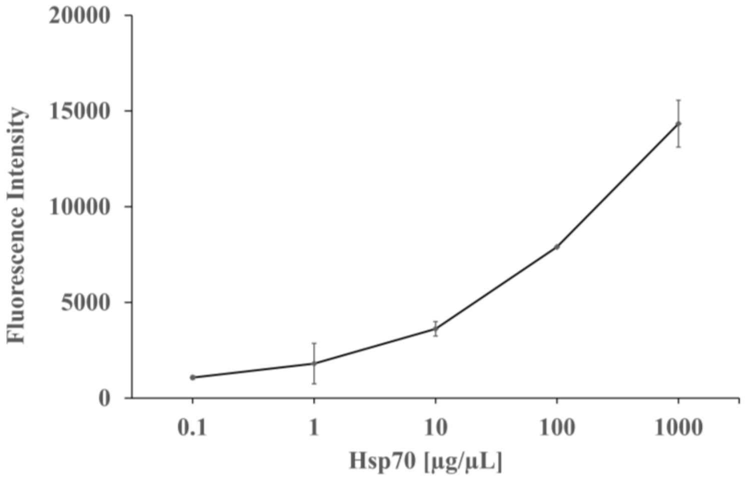

Figure S19. HSc-9.1 used as a capture molecule in a sandwich ELISA. Immobilization of 3‘-biotinylated-HSc-9.1 to streptavidin coated black plates and incubation with varying concentrations of Hsp70 followed by detection with anti-Hsp70 antibody labelled with Alexafluor.

2.15 **Specificity of HSc-9.1 aptamer against His-tagged proteins**

To test the specificity of the truncated HSc 9.1 aptamer against alternative His-tagged proteins, the binding affinity was measured by two methods- a) BLI – using Nickel coated sensors and b) Nickel-coated plate immunoassay. In the Nickel BLI assay, the concentration of HSc 9.1 was varied from 0-12.8 μM and the response was recorded with three different proteins namely, His-tagged CPA, His-tagged NPA and His-tagged SUMO. In all cases, the response of each was subtracted from the buffer controls. (Fig. S20a). For the fluorescence-based plate assay, the same His-tagged proteins were immobilised onto the nickel coated plate and then incubated with 0-3200 nM of 5'-6-FAM-labelled HSc 9.1 aptamer. The fluorescence intensity of any bound aptamers was measured using a Tecan microplate reader. Immobilised His-tagged Hsp70 and bound Alexa-fluor anti-Hsp70 antibody was used as a positive control with the binding buffer only considered for background subtraction. (Fig. S20b)

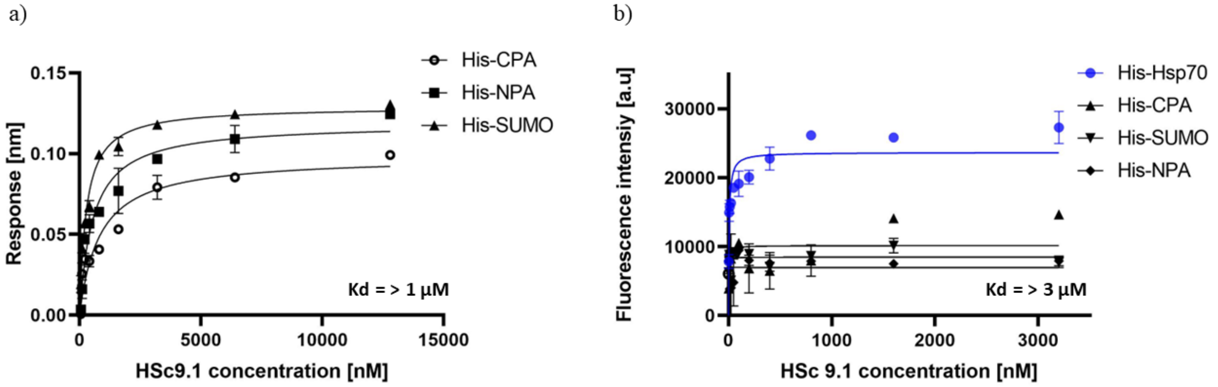

Fig. S20 HSc-9.1 aptamer specificity results towards three alternative His-tagged proteins. (a) The corresponding binding curves for the proteins as determined by BLI, different HSc-9.1 aptamer concentrations were used as analyte and the steady state binding affinity [K_d] was calculated. (b) The corresponding binding curves for the alternative His-tagged proteins as determined by the fluorescent plate-based binding assay. The raw fluorescent data were exported into Graph Prism and the binding affinity [K_d] of the aptamer against each protein was calculated. Error bars represent the average values of 2 independent experiments.

2.16 **Specificity of HSc-9.1 aptamer against Streptavidin binding peptide (SBP)-tagged Hsp70**

In-order to test the specificity of HSc-9.1 against Hsp70 with a different tag, the aptamer was incubated with Hsp70 that was tagged with a streptavidin binding protein (SBP) tag. 50 nM of SBP-tagged Hsp70 was immobilised onto streptavidin sensors and then incubated with HSc-9.1 aptamer ranging in concentration from 0-1000 nM. The response data obtained from BLI was analyzed by Graph Prism (Fig. S21).

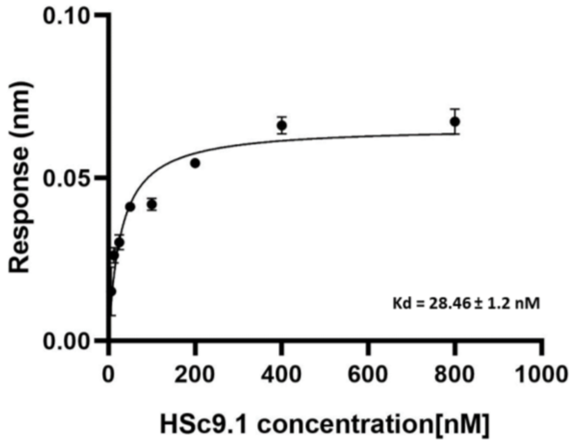

Fig. S21 Binding affinity results of HSc-9.1 aptamer against the SBP-tagged Hsp70. The graph shows the corresponding binding curve as determined by BLI, different aptamer concentrations were used as analyte and the steady state binding affinity [K_d] was calculated. The raw fluorescent data were exported into Graph Prism and the binding affinity [K_d] calculated. Error bars represent the average values of 2 independent experiments.

2.17 **Specificity of scrambled HSc-9 aptamer against His-tagged Hsp-70**

To test the sequence specificity, different scrambled aptamers (Table S3) were designed and their respective binding affinities were measured by the BLI and the fluorescent plate-based assay. Firstly, the A* modified scramble aptamers were synthesized by PEX method as described previously in the enzymatic synthesis section 2.7. His-tagged Hsp70 was immobilised onto

Nickel sensors and then incubated with the scrambled HSc-9 aptamers ranging in concentration from 0-800 nM. The response data obtained from BLI was analyzed by Graph Prism (Fig. S22). Both assays were performed as mentioned in the main manuscript section.

Supplementary Table S3: List of scrambled HSc-9 sequences:

Scramble sequence ID	Sequence 5'→ 3'
Scr1	GCAGCAGAGATAGACGCTA TCGA*TGA*CTA*TA*A*TTCCCA*TCTA*TCA*TCCA*A*TGGCA*TA*GCTGGCA*C
Scr2	GCAGCAGAGATAGACGCTA CCCCTTA*A*TA*A*A*A*A*CA*TA*CGGTCCA*TTCCCGTCGTA*CGA*TTGA*TTG
Scr3	GCAGCAGAGATAGACGCTA CCCCTTA*A*TA*A*A*A*A*CA*TA*CGGTCCA*TTCCCGTCGTA*CGA*TTGA*TTG

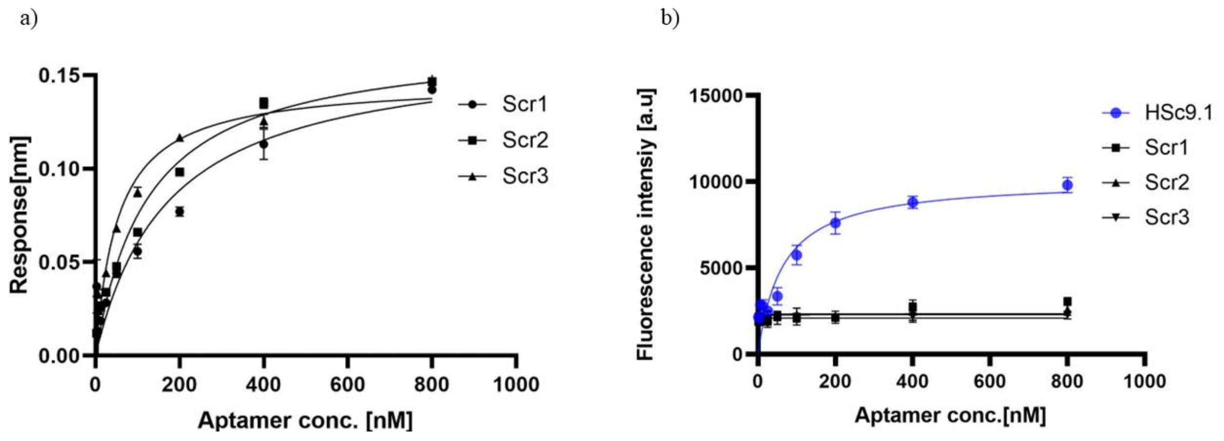

Fig. S22 Scrambled HSc-9 aptamer binding affinities towards His-tagged Hsp70. (a) The corresponding binding curves for the scrambled sequences as determined by BLI, different oligo concentrations were used as analyte and the steady state binding affinity [K_d] was calculated. (b) The corresponding binding curves as determined by the fluorescent plate-based binding assay. The raw fluorescent data were exported into Graph Prism and the binding affinity [K_d] of each

scrambled sequence was calculated. Error bars represent the average values of 2 independent experiments

Supplementary References

1. Seela, F.; Peng, X., Pyrrolo[2,3-d]pyrimidine β-L-Nucleosides Containing 7-Deazaadenine, 2-Amino-7-deazaadenine, 7-Deazaguanine, 7-Deazaisoguanine, and 7-Deazaxanthine. *Collection of Czechoslovak Chemical Communications* **2006,** *71*.
