# Supplementary material for: The selection of a hydrophobic 7-phenylbutyl-7-deazaadenine-modified DNA aptamer with high binding affinity for the Heat Shock Protein 70
Source: Commun Chem. 2023 Apr 6;6:65. doi: 10.1038/s42004-023-00862-0 (PMC10079658; doi:10.1038/s42004-023-00862-0)
Supplement: Supplementary file 3 — Description of Additional Supplementary File [file 42004_2023_862_MOESM3_ESM.pdf]

Description of Additional Supplementary File

File name: Supplementary Data 1

Description: NMR data
