# Supplementary material for: The selection of a hydrophobic 7-phenylbutyl-7-deazaadenine-modified DNA aptamer with high binding affinity for the Heat Shock Protein 70
Source: Commun Chem. 2023 Apr 6;6:65. doi: 10.1038/s42004-023-00862-0 (PMC10079658; doi:10.1038/s42004-023-00862-0)

^{1}H NMR (500.0 MHz, DMSO-d_6), compound 2 (dA[EEPh])

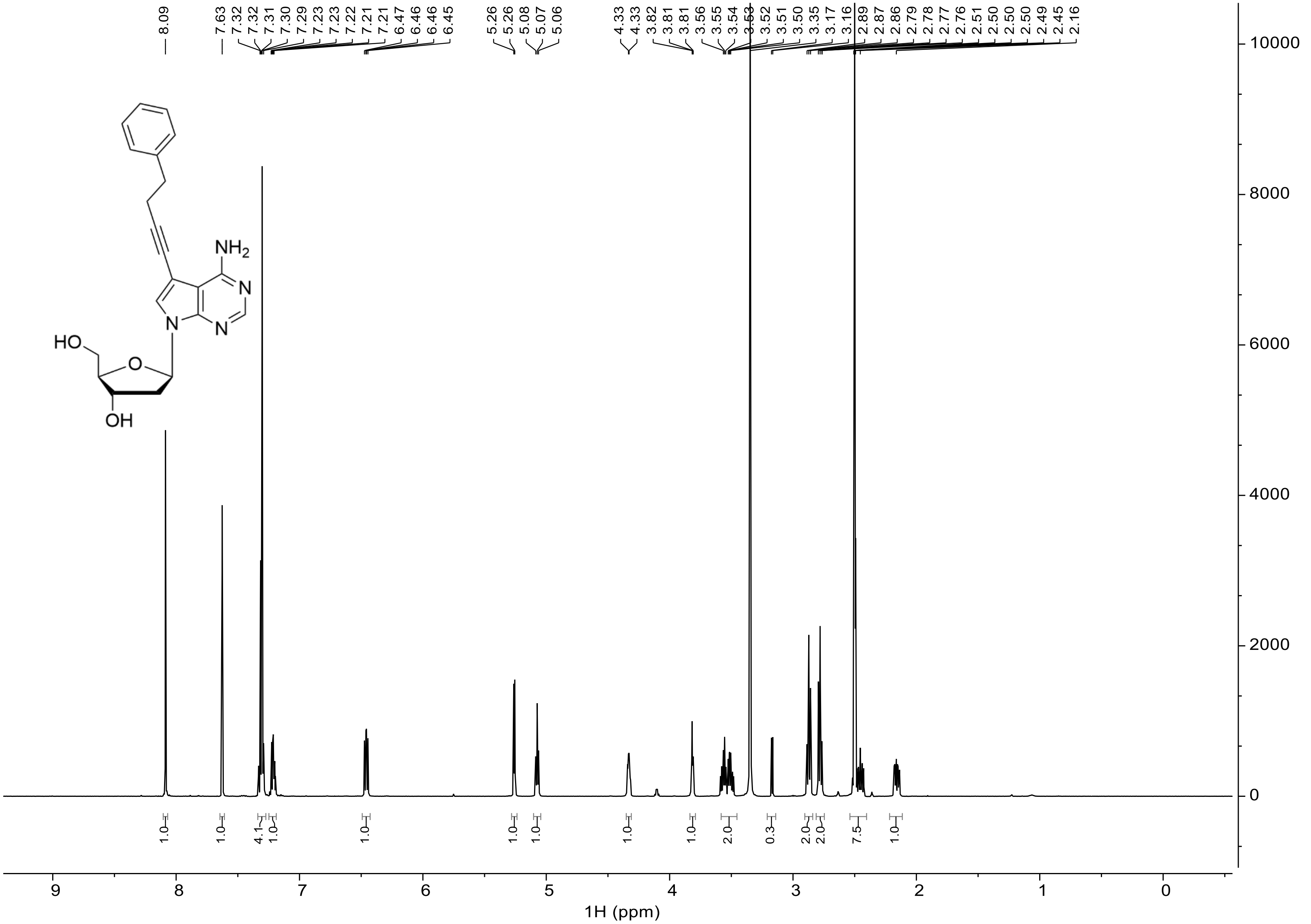

^{13}C NMR (125.8 MHz, DMSO-d_6), compound 2 (dA^{EEPh})

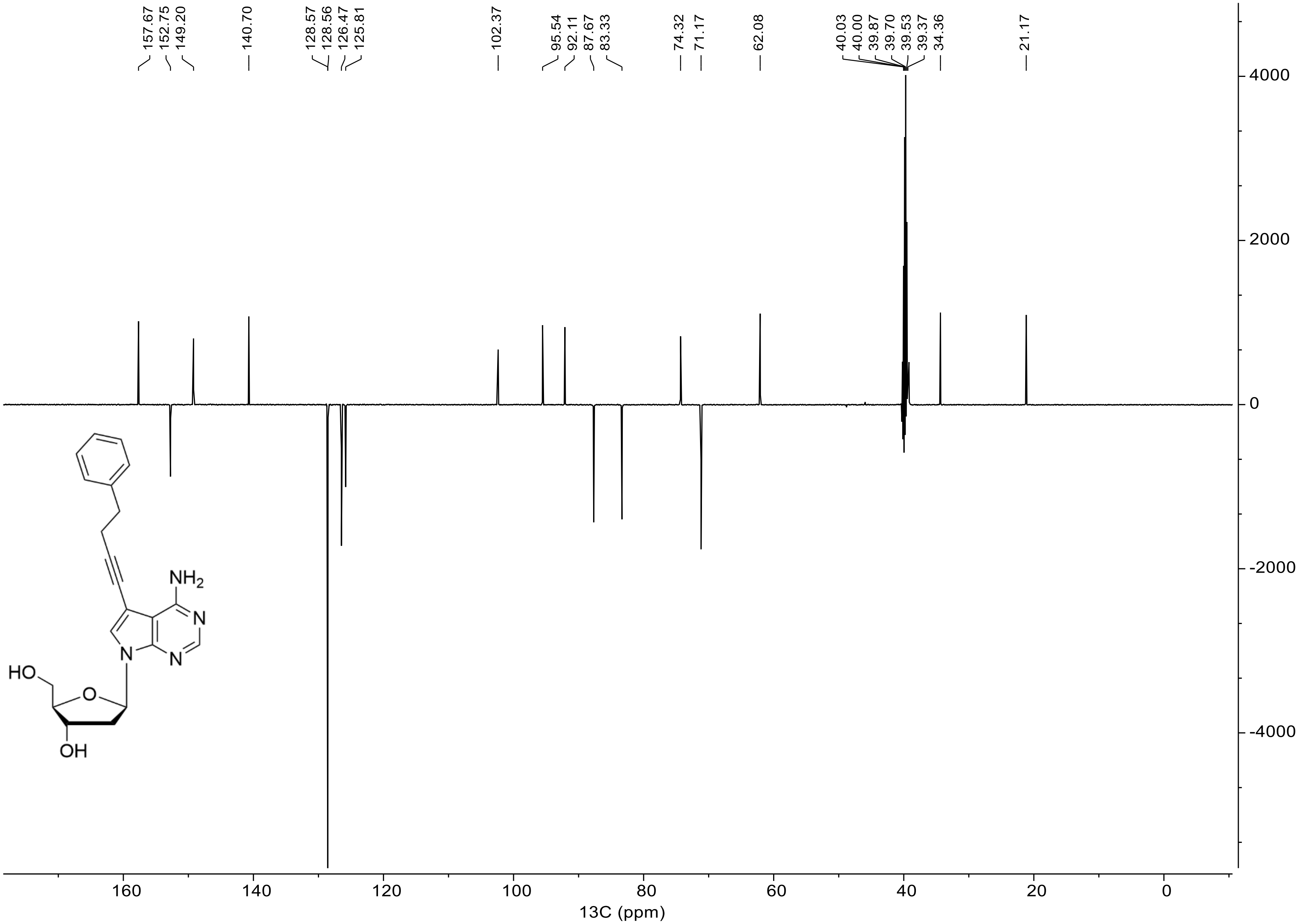

^{1}H NMR (500.0 MHz, DMSO-d_6), compound 3 (dA^{BuPh})

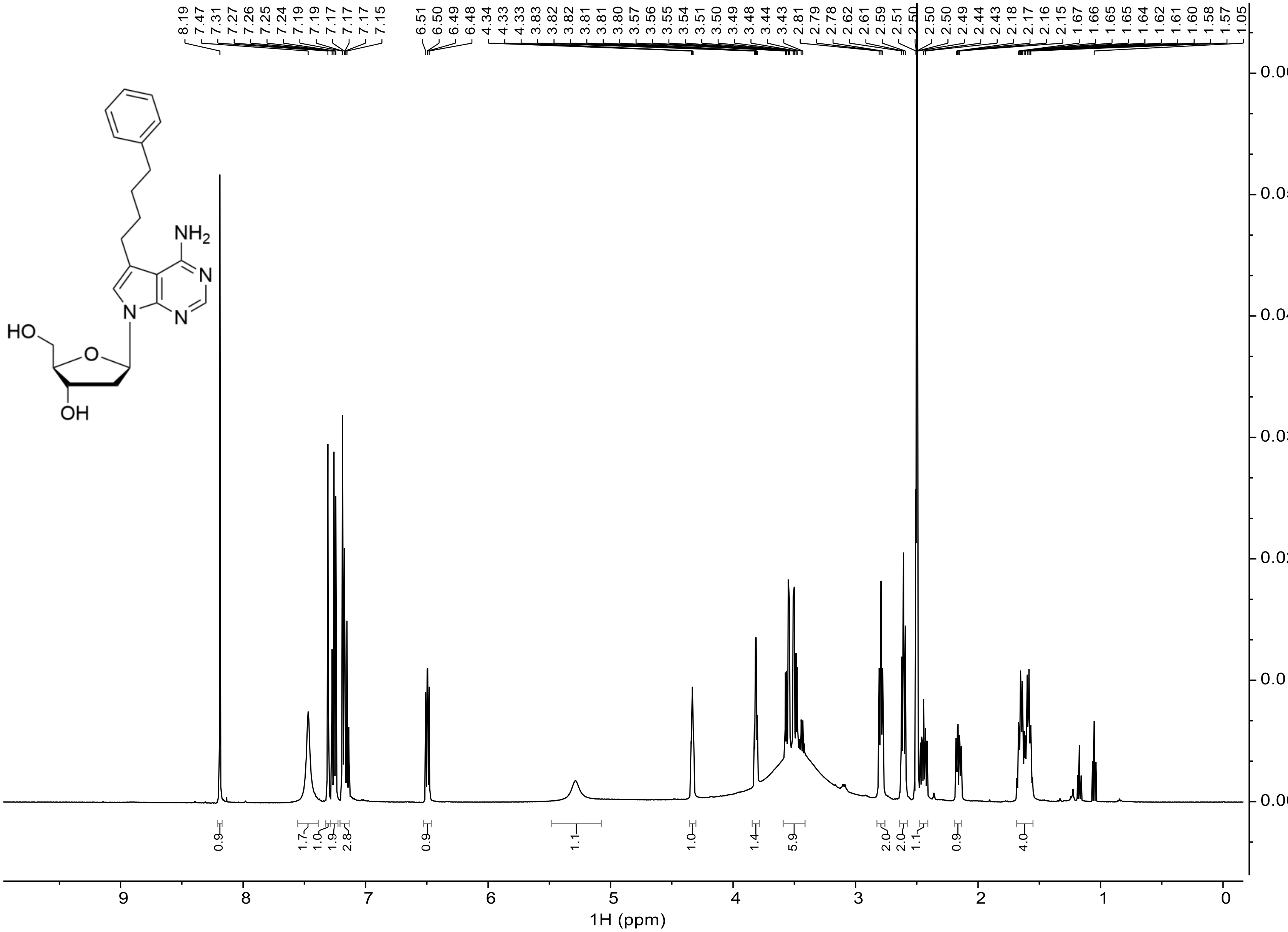

^{13}C NMR (125.8 MHz, DMSO-d_6), compound 3 (dA^{BuPh})

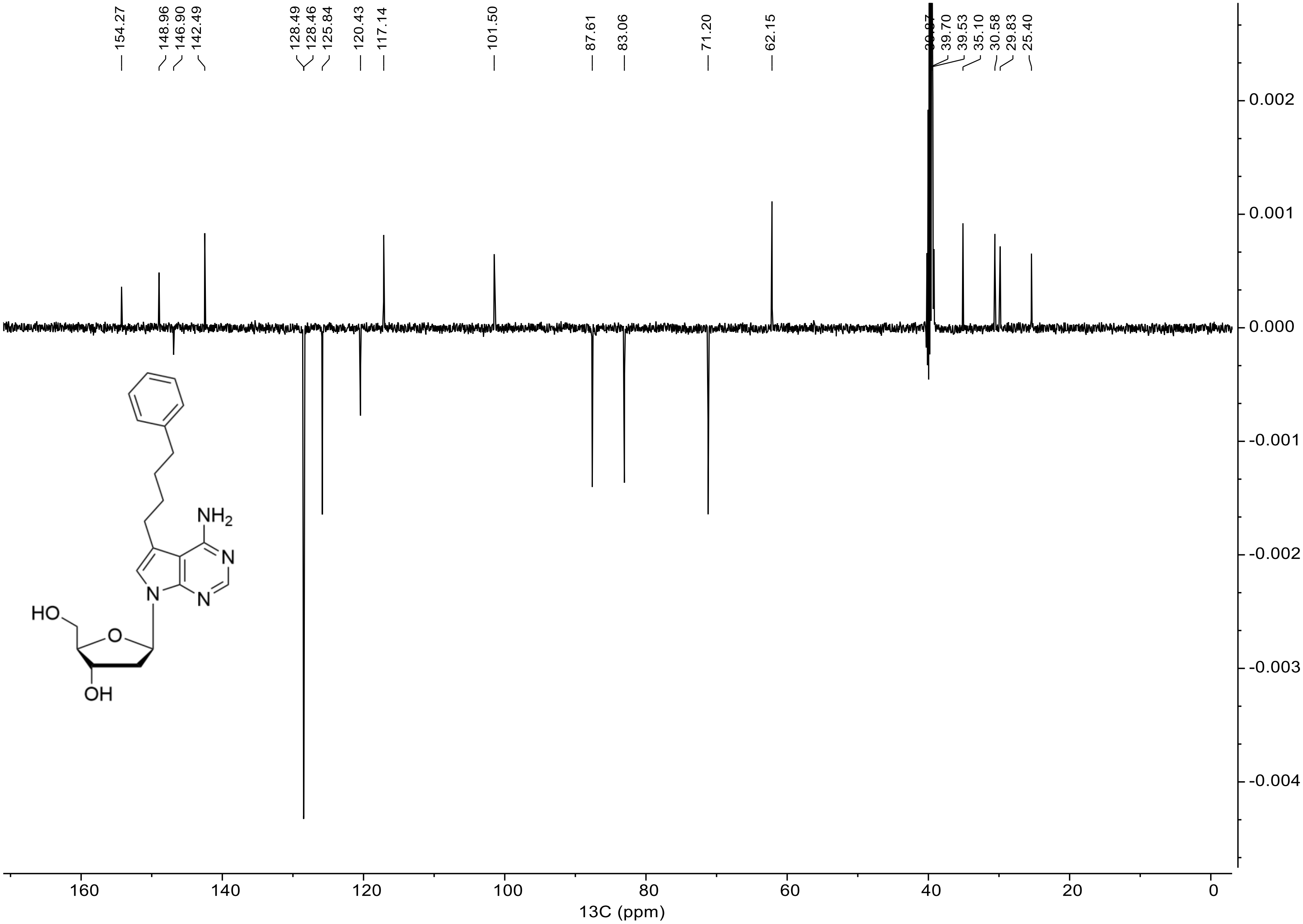

^{1}H NMR (500.0 MHz, CD_3OD), compound 4 (dA^{BuPh}TP)

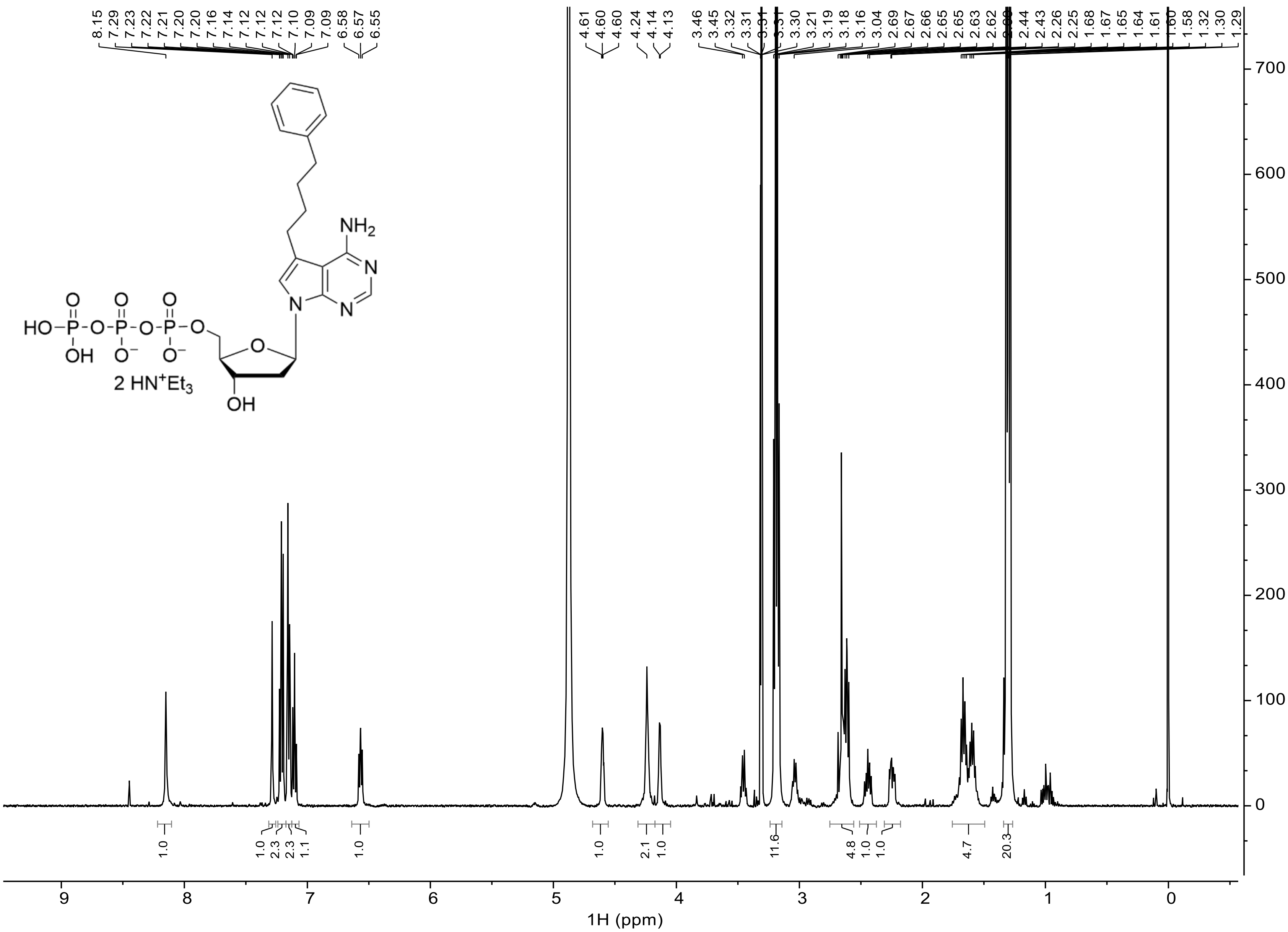

^{13}C NMR (125.8 MHz, CD_3OD), compound 4 (dA^{BuPh}TP)

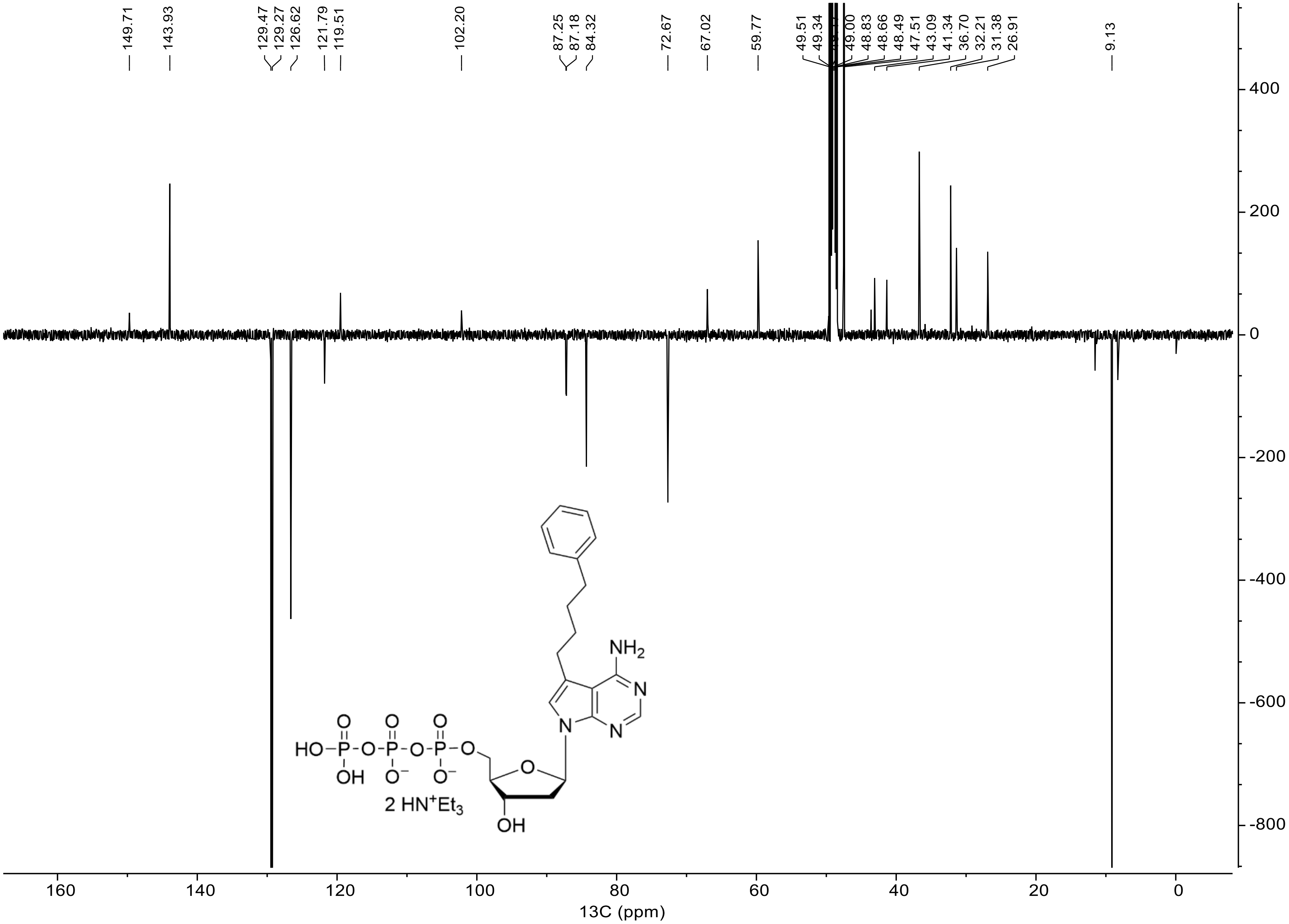

$^{31}P\{^1H\}$ NMR (202.4 MHz, CD_3OD), compound 4 (dA^{BuPh}TP)

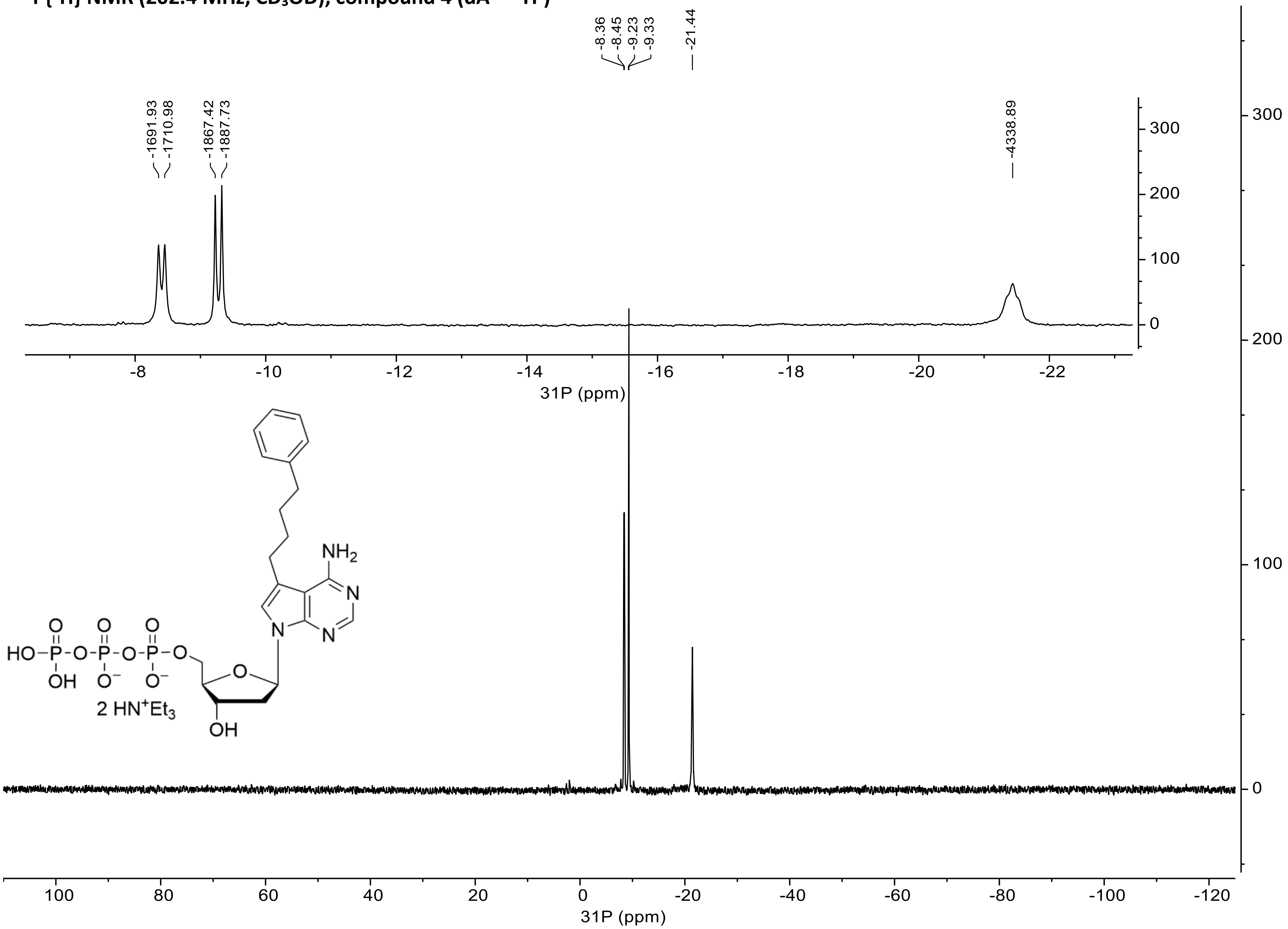

^{1}H NMR (500.0 MHz, DMSO-d_6), compound 5

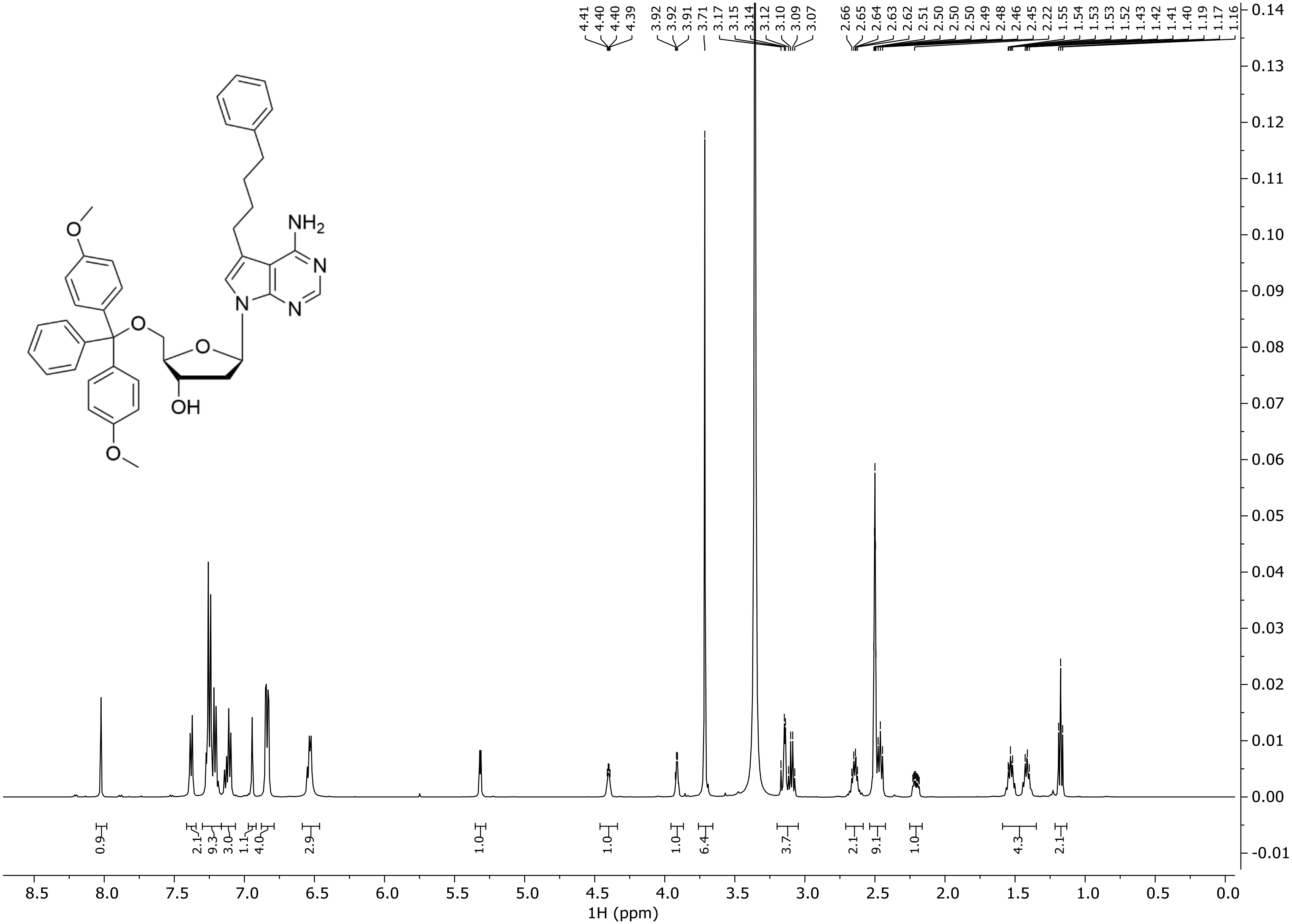

^{13}C NMR (125.8 MHz, DMSO-d_6), compound 5

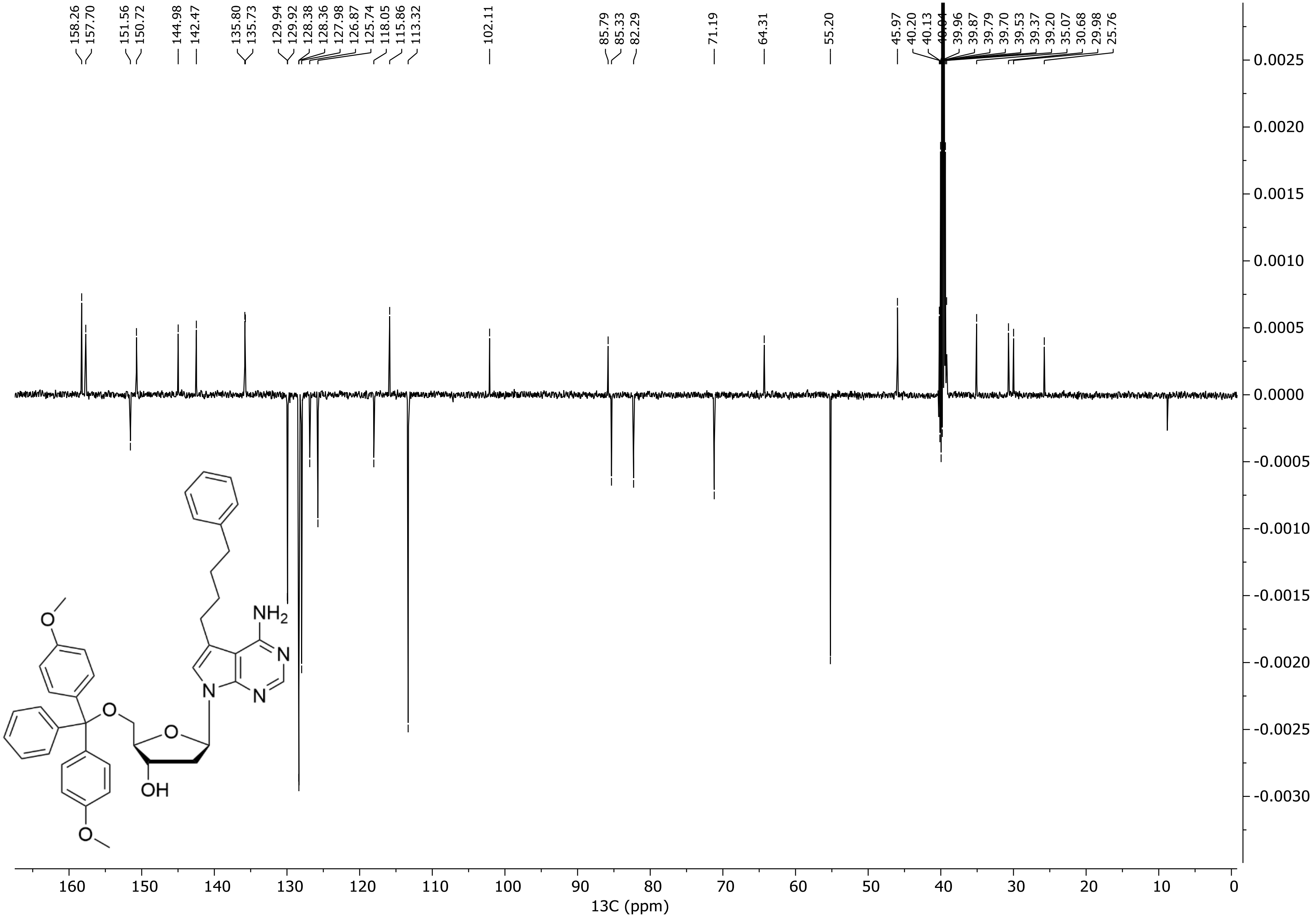

^{1}H NMR (500.0 MHz, DMSO-d_6), compound 6

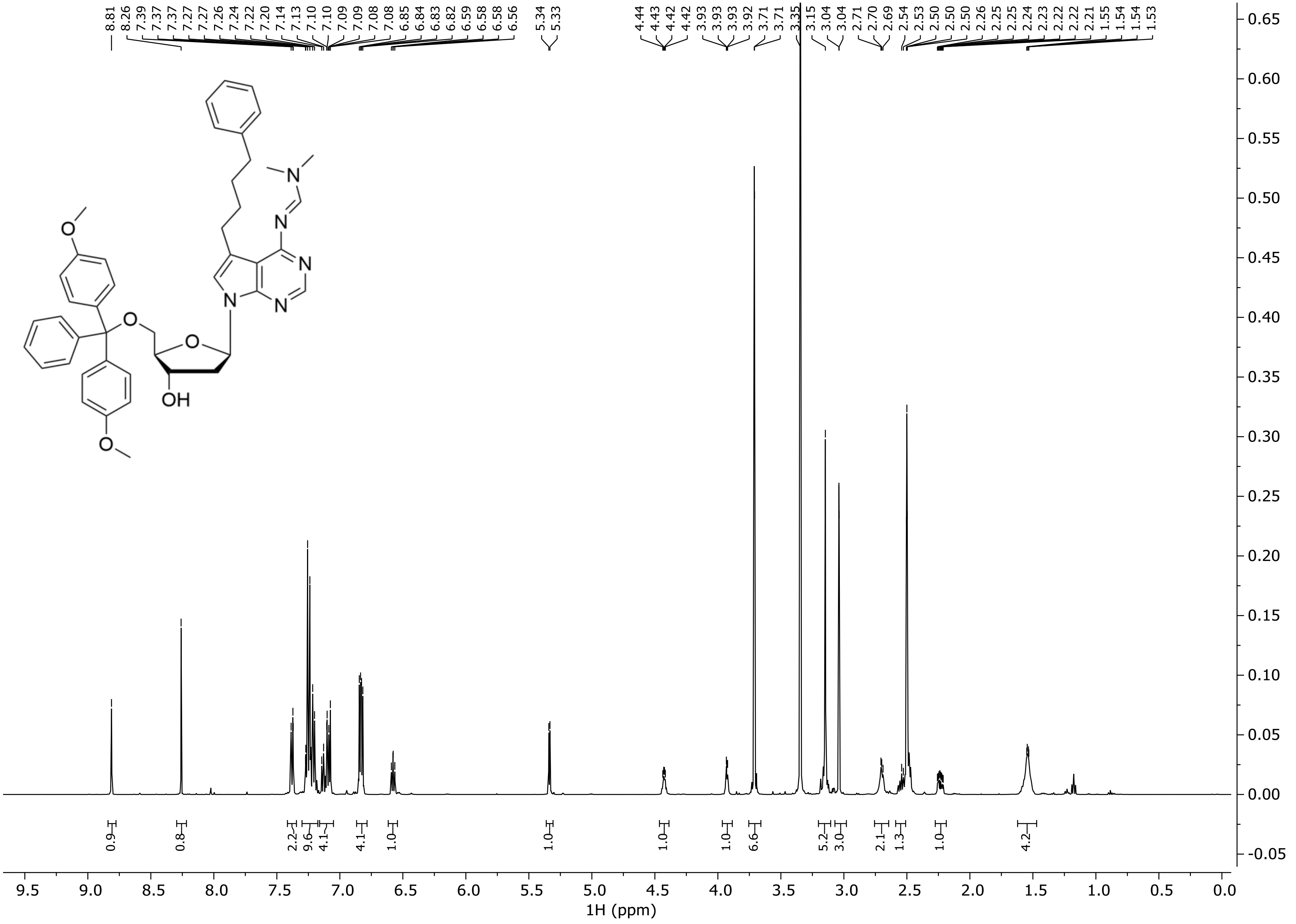

^{13}C NMR (125.8 MHz, DMSO-d_6), compound 6

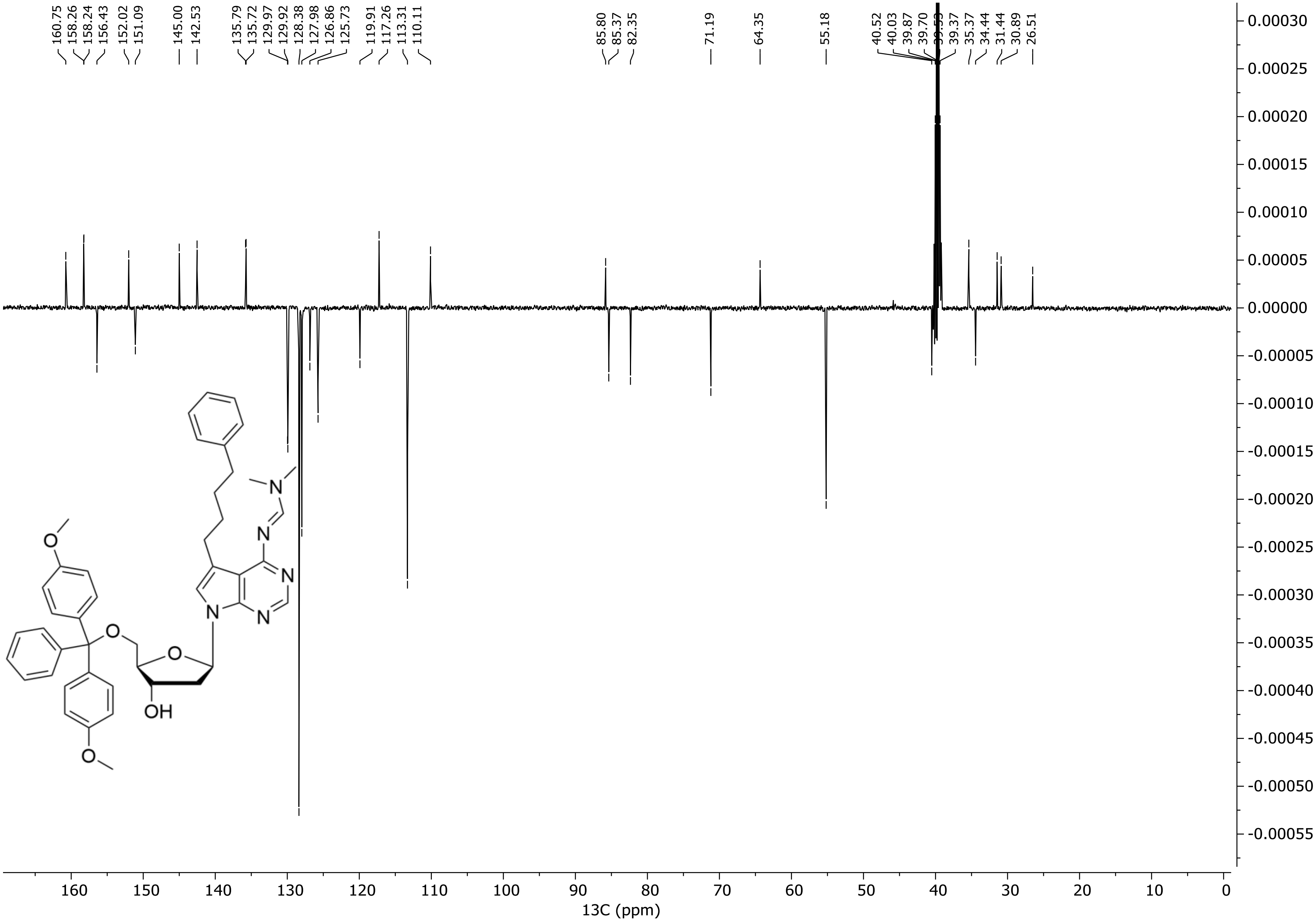

^{1}H NMR (500.0 MHz, CD_3CN), compound 7

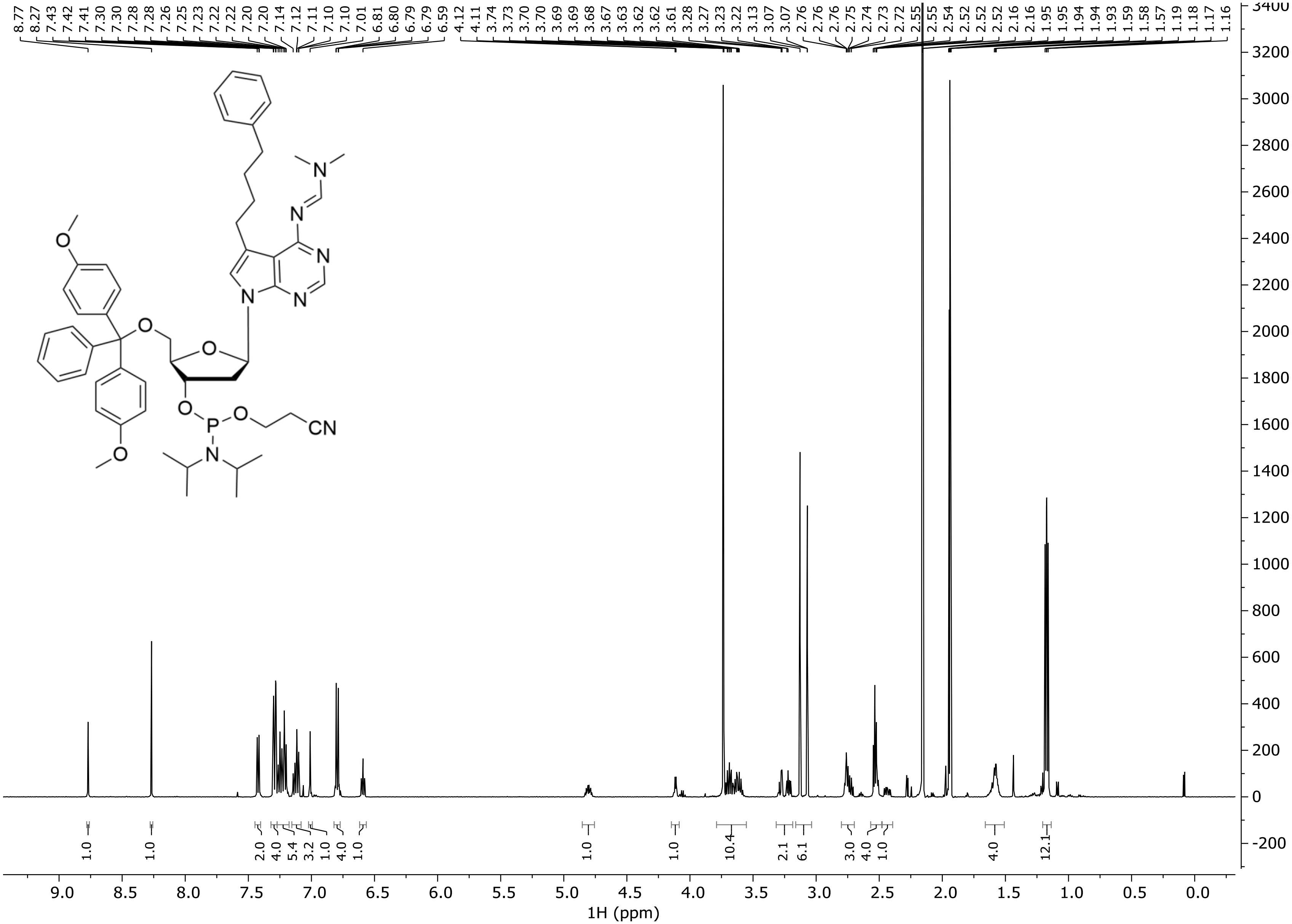

^{13}C NMR (125.8 MHz, CD_3CN), compound 7

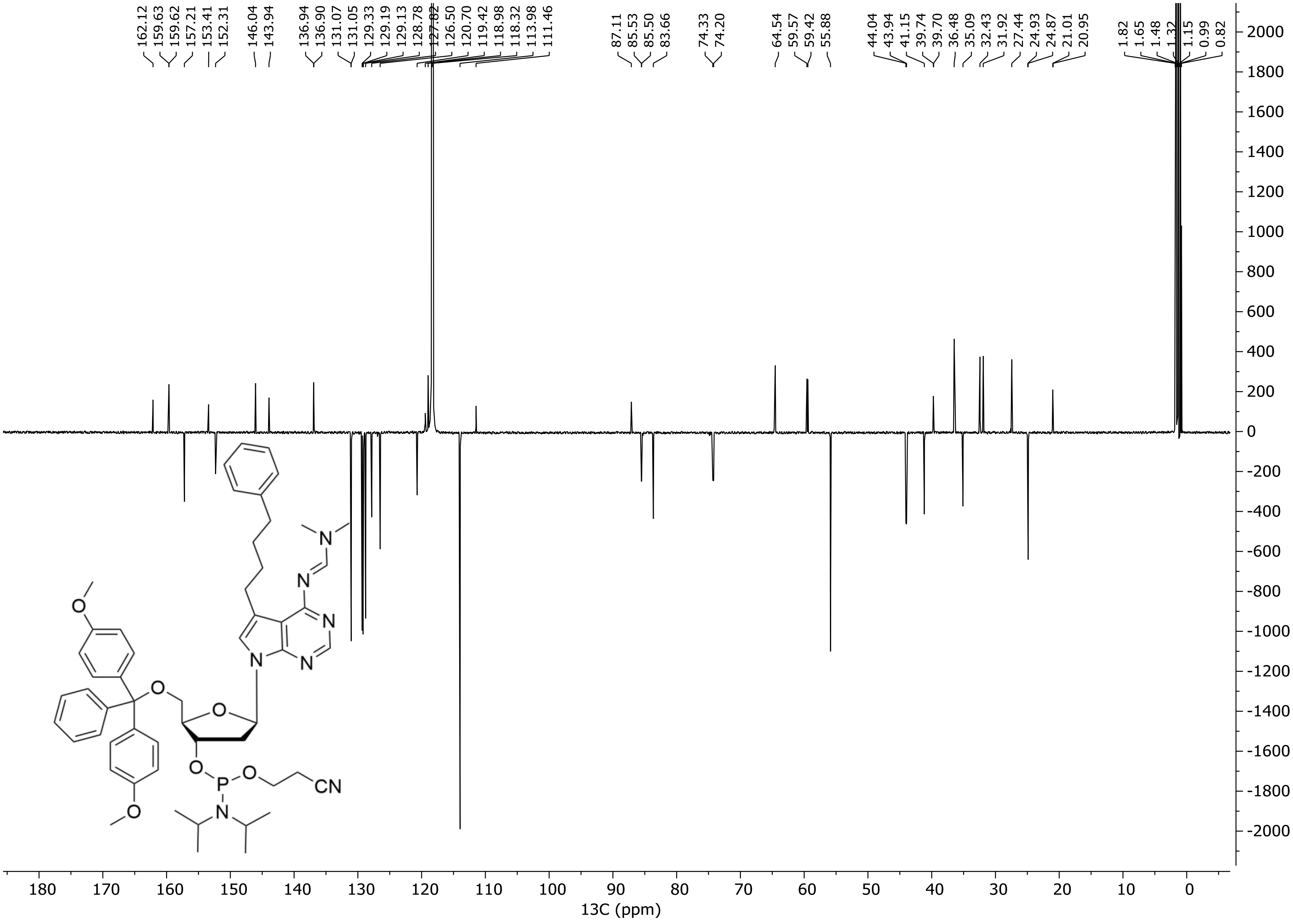

^{31}P NMR (202.4 MHz, CD_3CN), compound 7

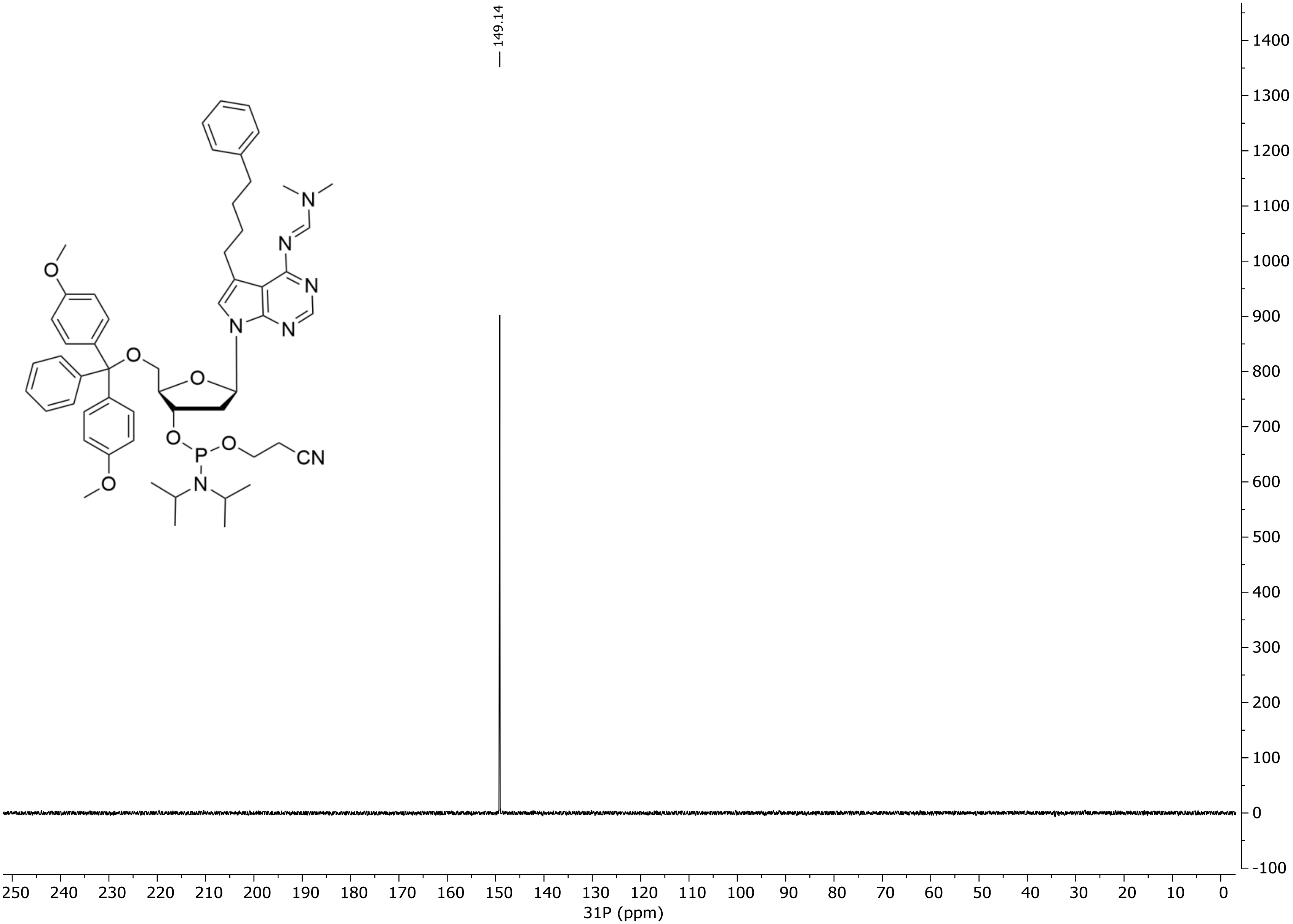

Supplement: Supplementary file 4 — Supplementary Data 1 [file 42004_2023_862_MOESM4_ESM.pdf]
